# Large language model assistance for report-based carotid plaque AHA classification: a multi-reader study

**DOI:** 10.1186/s13244-026-02421-2

**Published:** 2026-10-16

**Authors:** Mengfei Wang, Yixiao Tang, Yulu Yang, Yifan Tu, Zhongzheng Cao, Runjianya Ling, Kaiyue Sun, Jian Ma, Zhicheng Zhang, Ruipeng Zhang, Xiaoer Wei, Dan Wang, Yuehua Li

**Affiliations:** 1 Department of Radiology, Shanghai Sixth People’s Hospital Affiliated to Shanghai Jiao Tong University School of Medicine, Shanghai, China; 2 School of Information and Intelligent Science, Donghua University, Shanghai, China; 3 Department of Radiology, Shanghai East Hospital, Tongji University School of Medicine, Shanghai, China; 4 Department of Radiology, The Affiliated Huaian No.1 People’s Hospital, Huaian, China

**Keywords:** Carotid arteries, Plaque (atherosclerotic),, Magnetic resonance imaging, Artificial intelligence, Stroke

## Abstract

**Objectives:**

To evaluate whether large language model assistance improves radiologists’ agreement with a text-based reference standard in inferring the modified American Heart Association carotid plaque classification from free-text MRI reports, and whether a lightweight model can perform well under criteria-guided prompting.

**Materials and methods:**

This retrospective study included 433 patients from three institutions. In a report-based reader study, four radiologists classified MRI reports with and without large language model assistance. In standalone evaluation, a rule-based baseline and 10 models were compared under naive and criteria-guided prompting. The reference standard was expert consensus based on the report text.

**Results:**

Among 433 patients (mean age, 65.9 years ± 13.4; 248 males), AI assistance improved six-class agreement with the text-based reference standard. At the vessel level, junior radiologists improved from 61.75% to 83.12% (Qwen3-8B) and 87.75% (DeepSeek-R1); senior radiologists from 77.00% to 87.50% and 92.25%, respectively (all Holm-corrected *p* < 0.001). Patient-level agreement increased from 41.0% to 78.8% (junior) and from 59.8% to 87.8% (senior) with DeepSeek-R1. Agreement increased (quadratic-weighted *κ*: 0.231 without AI vs 0.775 with DeepSeek-R1). In standalone evaluation, criteria-guided prompting improved Qwen3-8B agreement from 38.91% to 81.52% (95% CI: 78.87–84.06%), remaining 5.89 percentage points below DeepSeek-R1 (87.41%; 95% CI: 85.10–89.61%).

**Conclusion:**

In this report-based classification setting, large language model assistance improved radiologists’ agreement with the text-based reference standard, with larger gains in junior radiologists. When prompts explicitly encoded AHA criteria, an 8B local candidate model performed well, supporting its potential use as a standardization aid for text-based workflows.

**Key Points:**

***Question*** Report-based modified AHA classification of carotid plaques is experience-dependent and difficult to standardize, as mapping multi-sequence imaging findings to classification conclusions imposes substantial cognitive load.

***Findings*** Large language model assistance improved agreement with a text-based reference standard and raised inter-reader consistency (weighted κ 0.231–0.775), narrowing the junior–senior gap.

***Critical relevance statement*** Embedding explicit AHA criteria in the prompt let an 8B model approach 671B-model agreement (38.91–81.52%), supporting standardized text-based workflows without fine-tuning.

**Graphical Abstract:**

**Figure:**
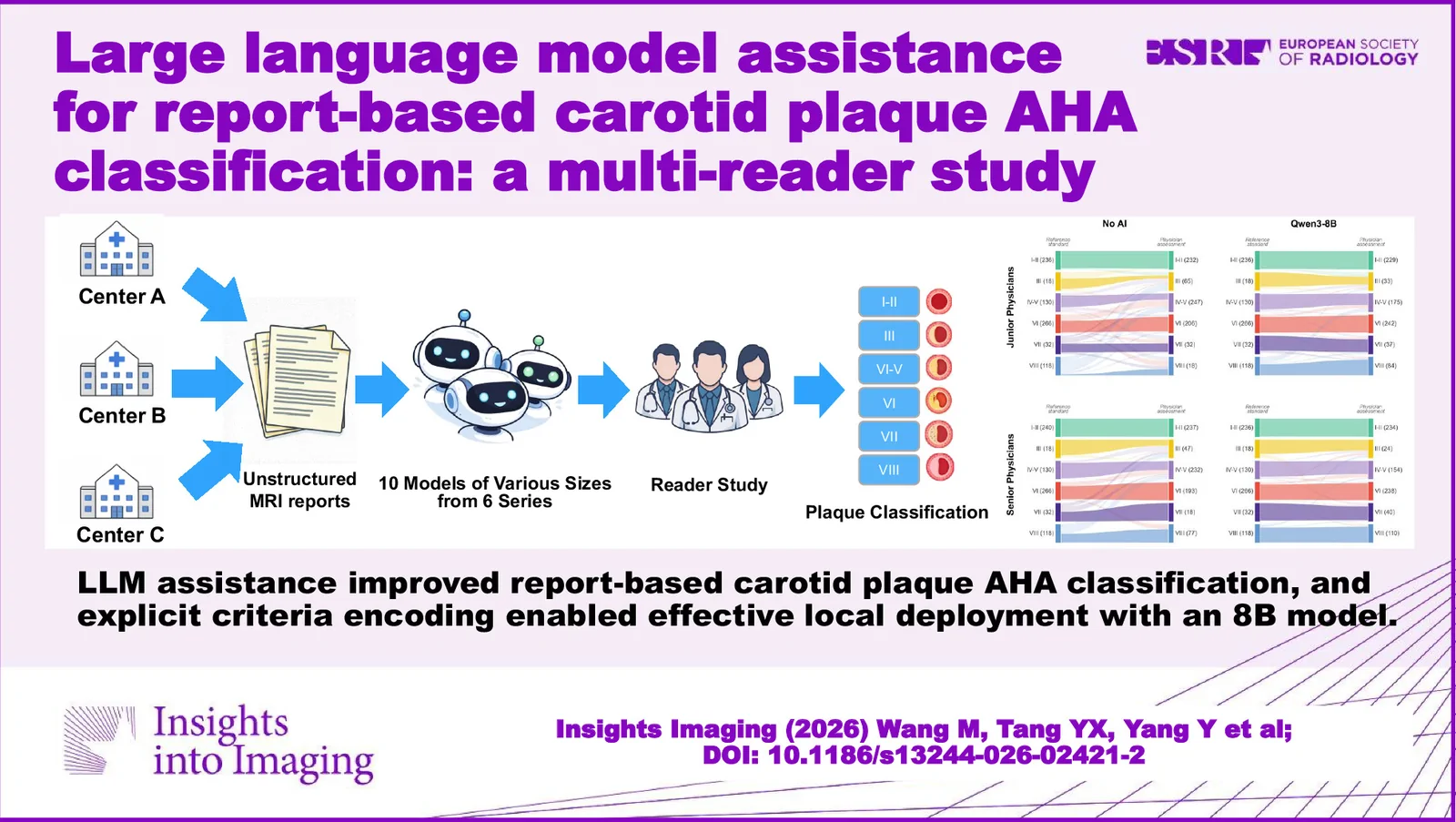

## Introduction

Carotid atherosclerotic plaques are present in approximately one in five patients with transient ischemic attack or stroke [1–3]. Identifying vulnerable plaques with high-risk features such as intraplaque hemorrhage is critical for the prevention of cerebrovascular events [4, 5]. The modified American Heart Association (AHA) classification based on high-resolution magnetic resonance imaging (HRMRI) provides a standardized risk-stratification framework for plaque composition [6–9], categorizing plaques into six operational classes according to tissue components (lipid-rich necrotic core, fibrous tissue, calcification, hemorrhage, etc.) and surface integrity based on multi-sequence MRI signal characteristics. Type VI plaques (with intraplaque hemorrhage and/or surface disruption) are associated with significantly increased ischemic risk [5, 10, 11]. However, standardized implementation of the AHA classification remains limited [12]: unlike luminal stenosis assessment, which involves a single quantitative metric, this process requires physicians to integrate multi-sequence, multi-finding information and apply hierarchical criteria to determine the final type, imposing a substantial cognitive load. Prior studies have shown that radiologists demonstrate high consistency in identifying individual imaging findings (e.g., signal characteristics, fibrous cap status), but considerably lower consistency when integrating these findings into a composite classification conclusion [13, 14], suggesting that this cognitive mapping from imaging findings to classification conclusions constitutes an experience-dependent bottleneck [15, 16].

Because this bottleneck occurs primarily at the step of translating report descriptions into a standardized label, report-based approaches can intervene directly at that stage. However, model performance remains dependent on the completeness and consistency of the original reports, which may vary across readers and institutions. Recent studies have shown that large language models (LLMs) can extract structured information from free-text radiology reports and perform standardized classification tasks across multiple domains [17–20]. However, general-purpose models remain difficult to deploy clinically because of privacy concerns, computational cost, and outputs that may be difficult to map consistently to predefined clinical criteria [21, 22]. To date, no study has systematically evaluated whether LLM assistance can improve radiologists’ report-based inference of carotid plaque AHA classification.

To address this, we evaluated a task-specific prompting strategy that explicitly embeds the modified AHA criteria into the model instructions and decomposes the classification into two auditable steps—mapping signal descriptions to plaque components, then applying hierarchical criteria to assign the final type (criteria-guided prompting (CGP)). Rather than introducing a new model architecture, this was intended as a pragmatic way to align outputs with established clinical rules, make the basis for classification easier to review, and reduce dependence on large annotated datasets. This study was designed as a report-based text-to-label mapping evaluation, in which imaging findings described in free-text reports were mapped to standardized AHA classifications. This retrospective study had two aims. First, in a report-based reader study, we evaluated whether LLM assistance improves radiologists’ six-class agreement with a text-based reference standard, with Type VI detection sensitivity as a key secondary endpoint and agreement with the reference standard as an additional performance measure. Second, we assessed whether an open-source small-parameter model can approach the classification performance of a much larger model when the prompt explicitly encodes the modified AHA criteria.

## Materials and methods

### Study design and ethics statement

This retrospective study was approved by the ethics committees of Shanghai East Hospital affiliated with Tongji University, Huai’an First People’s Hospital, and Shanghai Sixth People’s Hospital Affiliated to Shanghai Jiao Tong University School of Medicine (institutional review board approval No. 2024-KY-203 [K]), and patient informed consent was waived in accordance with the Declaration of Helsinki. We queried consecutive patients who underwent carotid HRMRI (scanners from Siemens Healthineers, Philips Healthcare, GE Healthcare, and United Imaging; detailed acquisition parameters provided in Table S1) at the three institutions between January 2023 and September 2024.

**Fig. 1:**
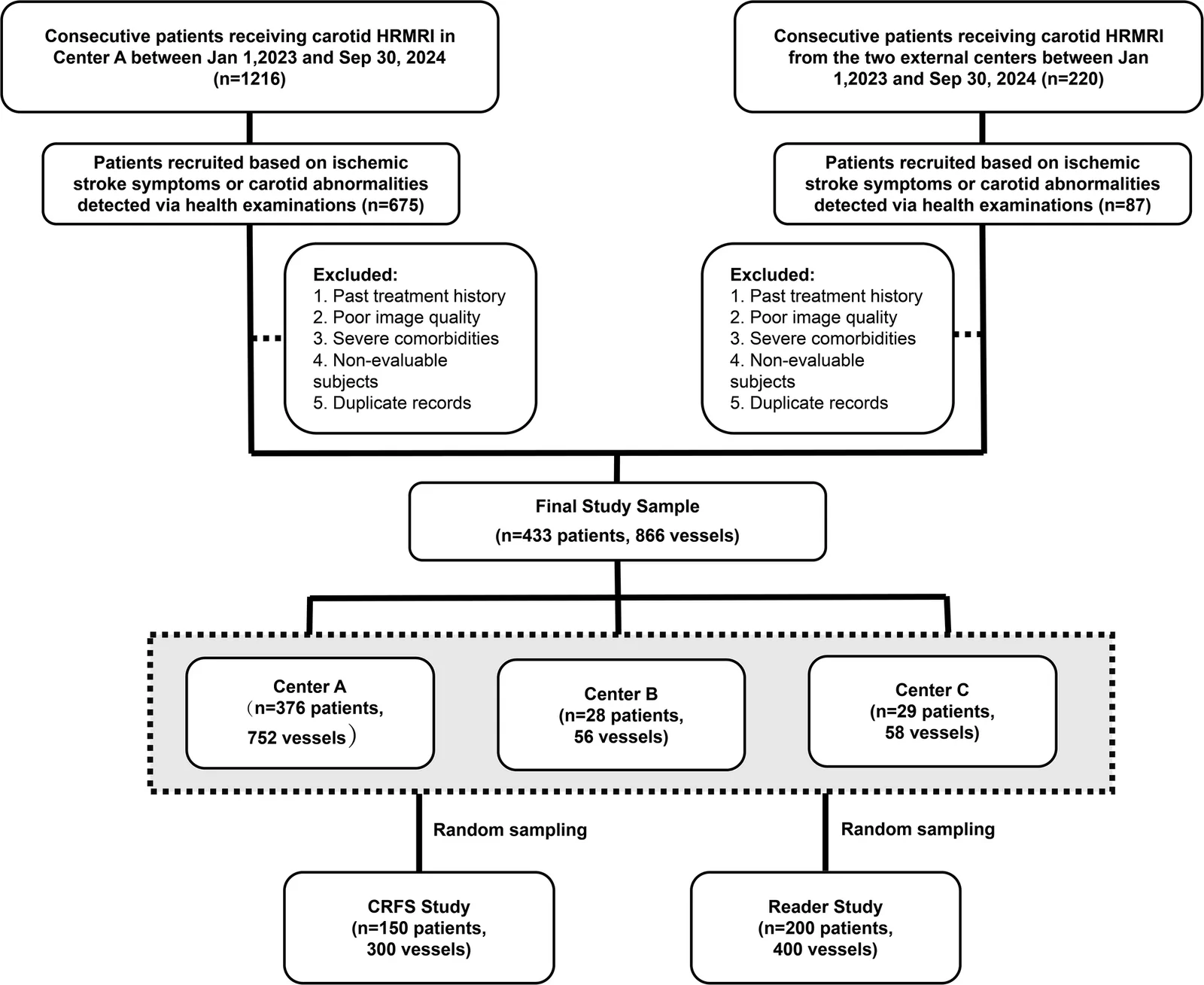
Patient selection flow diagram. A total of 762 patients who underwent carotid high-resolution magnetic resonance imaging (HRMRI) at three centers were screened. After applying exclusion criteria (prior carotid endarterectomy or stenting, severe artifacts, severe comorbidities, bilateral occlusion or normal vessels, and duplicate records), 433 patients with 866 carotid vessels were included for primary analyses. The cohort was distributed across Center A (*n* = 376), Center B (*n* = 28), and Center C (*n* = 29). A stratified random sample of 200 patients (400 vessels) was selected for the reader study

**Fig. 2:**
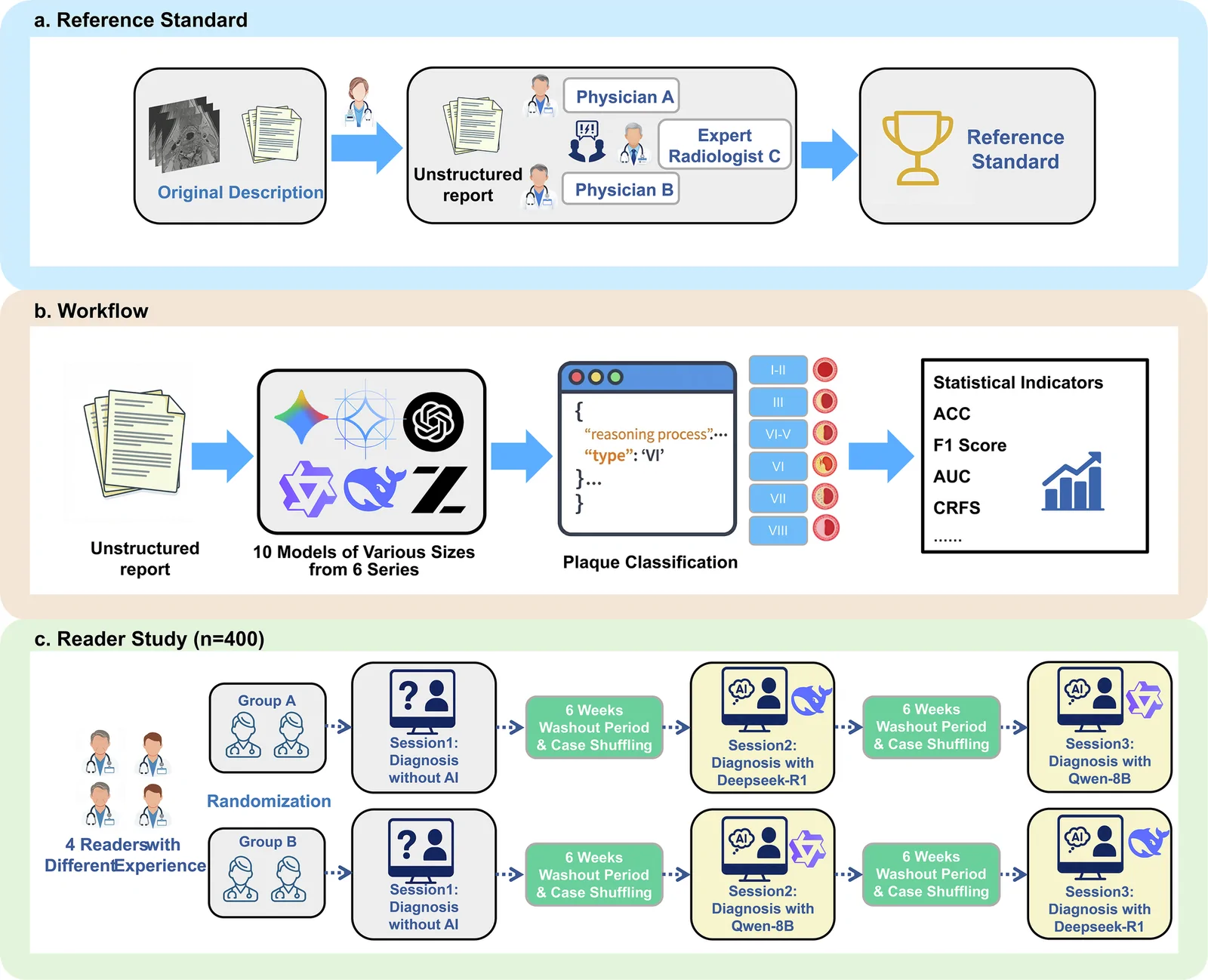
Overall study workflow and experimental design. **a** Reference standard establishment. De-identified free-text HRMRI reports were reviewed by senior neurovascular radiologists to establish a text-based consensus modified AHA classification. **b** Standalone AI performance evaluation. The study compared a rule-based baseline against 10 large language models (LLMs) across different parameter scales under naive and criteria-guided prompting strategies. **c** Human–AI collaboration reader study. A fixed sequence with the unaided condition first and balanced ordering of AI-assisted conditions with a 6-week washout period was employed to evaluate the agreement performance of four radiologists (two junior, two senior) with and without AI assistance

The initial query identified 762 patients who underwent imaging for ischemic stroke symptoms or abnormal carotid findings detected during screening. Patients with prior carotid intervention, severe image artifacts, severe systemic diseases, or bilateral vessels without plaque were excluded (selection flowchart shown in Fig. 1). The final cohort comprised 433 patients (unit of analysis: 866 carotid vessels). All HRMRI reports and data were de-identified on local hospital servers. Prompts were developed and optimized on an independent subset of 50 patients from Center A (non-overlapping with the 433 patients in the primary analysis); the main cohort of 433 patients from Centers A, B, and C was used for final performance validation. Center A contributed 376 patients (86.8%), whereas Centers B and C contributed 28 and 29 patients, respectively; therefore, this was a primary-center-dominant cohort with limited external sampling (overall study workflow shown in Fig. 2).

### Reference standard establishment

The reference standard was a text-based consensus reference standard, defined as “the most reasonable classification derivable from the given imaging report text according to modified AHA classification criteria.” This standard was designed to evaluate the consistency of cognitive mapping from report descriptions to standardized classification, rather than to directly reflect disease ground truth at the imaging or pathological level. After a radiologist with more than 5 years of experience extracted and de-identified carotid morphology descriptions, two readers independently performed the classification assessment, with high vessel-level inter-reader agreement (96.42%; Cohen’s κ = 0.95). Disagreements were resolved through consensus discussion, and any remaining unresolved cases were adjudicated by a third senior expert with 20 years of clinical experience. The overall workflow is shown in Fig. 2a.

### AI classification framework and prompting strategy

We designated the lightweight Qwen3-8B as the primary index test, DeepSeek-R1 (671B) as the performance upper-bound comparator, and a rule-based text extraction method as the lower-bound baseline. Additionally, we evaluated eight other LLMs of varying parameter scales as exploratory comparisons, including open-source general-purpose models, a medically fine-tuned model (MedGemma), and a commercial closed-source model (Gemini 2.5 Pro) (detailed model list provided in Table S2). For reporting guideline compliance, we referenced the Checklist for Artificial Intelligence in Medical Imaging (CLAIM) [23].

To align model outputs with predefined clinical rules, we used a task-specific prompting strategy that explicitly embedded the modified AHA classification criteria in the prompt (CGP). Briefly, this prompting strategy required criteria-guided two-stage reasoning: first, separately parsing the left and right carotid reports to map MRI signal descriptions to potential plaque components and structural features; and second, using the embedded modified AHA criteria, signal-characteristic tables, and key differential rules to sequentially exclude inapplicable types and determine the final AHA classification for each vessel. The detailed workflow is shown in Fig. 2b. Except for Gemini 2.5 Pro, which was accessed via API, all models were deployed on a local computing cluster equipped with NVIDIA GPUs. All models used temperature = 0.1 and maximum output length (max_tokens) = 8192 to minimize generation randomness and ensure reproducibility of classification results. To further assess output stability and cross-language consistency, methods for the Chinese–English bilingual evaluation and repeated-run stability testing of Qwen3-8B are described in Table S15.

### Report-based human–AI collaboration study and clinical reasoning fidelity

To evaluate report-based text-to-label mapping performance, four radiologists (two junior (4–5 years of experience), two senior (7–9 years of experience)), none of whom had dedicated neurovascular imaging subspecialty training, participated in a report-based reader study. All readers first completed baseline classification without AI assistance. In the subsequent AI-assisted phase, readers were assigned in a balanced order to two groups and performed AHA classification for 200 stratified randomly sampled patients based solely on report text, following either the Qwen3-8B→DeepSeek-R1 or DeepSeek-R1→Qwen3-8B sequence. A 6-week washout period was implemented between the two AI-assisted conditions.

This study employed a report-scenario simulation design: physicians were provided only de-identified free-text reports rather than original images, to purely quantify the benefit of AI assistance at the “report description → standardized classification” cognitive mapping stage. Six-class overall agreement was the prespecified primary endpoint, and Type VI detection sensitivity was the key secondary endpoint. The overall workflow is shown in Fig. 2c.

Clinical Reasoning Fidelity Score (CRFS, 1–5 scale) assessment was conducted in two stages. In this study, CRFS scoring was performed by three senior radiologists with 10–20 years of clinical experience. These radiologists were involved only in CRFS assessment and did not participate in prompt design, reference standard adjudication, model optimization, or the human–AI collaborative reader study. In Stage 1, three senior radiologists independently scored 200 cases to verify inter-rater reliability (ICC[1,2] = 0.917, 95% CI: 0.904–0.930). Based on these satisfactory reliability results, in Stage 2, one of the three raters described above independently completed formal CRFS scoring of AI reasoning texts for 150 patients (300 vessels). All raters were blinded to model identity, prompting strategy, and reference standard labels.

### Statistical analysis

Prespecified confirmatory comparisons included: (1) six-class agreement differences between AI-assisted and unassisted conditions (primary endpoint); (2) changes in Type VI detection sensitivity with AI assistance (key secondary endpoint). Remaining analyses (including experience gap change magnitude, CRFS scores, interpretation time, etc.) were reported as descriptive or exploratory analyses. Because this study was a retrospective consecutive cohort study, no formal a priori sample size or power calculation was performed. To assess the precision of the primary endpoint comparisons, we conducted a post hoc sensitivity analysis based on the paired exact McNemar test, calculating the minimum detectable improvement proportion and corresponding difference in agreement at 80% power with a two-sided α of 0.05 for a given number of discordant pairs. At the vessel level, the observed proportion of discordant pairs was 22.5–34.2%, corresponding to a minimum detectable difference of approximately 6–8 percentage points. At the patient level, the proportion of discordant pairs was 35.0–50.5%, corresponding to a minimum detectable difference of approximately 11–14 percentage points. The observed effect sizes for all confirmatory comparisons exceeded the corresponding minimum detectable thresholds. Given that each case included bilateral carotid arteries and the same reader provided classifications under multiple conditions, observations were not fully independent; accordingly, patient-level clustered bootstrap resampling (1000 iterations) was used to calculate 95% confidence intervals (CIs) in the standalone AI performance evaluation. In the reader study, we performed both vessel-level and patient-level agreement analyses: vessel-level treated left and right carotid arteries as independent laterality observations, while patient-level was defined as correct classification on both sides. Paired agreement comparisons for individual readers used exact McNemar tests; group-level primary comparisons between junior and senior physicians used logistic generalized estimating equation (GEE) models with patient-level clustering, with vessel-level models additionally adjusting for laterality, and within-group method comparisons additionally adjusting for reader identity. The session-order effect in the AI-assisted phase was evaluated as a post hoc analysis using a logistic GEE model, with classification correctness as the binary outcome and patient-level clustering. The model included an AI model, an AI-assisted session, reader identity, vessel side, and the interaction between the AI model and AI-assisted session to test whether the performance of different AI models was affected by the order of AI-assisted sessions. To evaluate the reproducibility of LLM outputs, we performed repeated-run stability experiments: Qwen3-8B was independently run three times under both NP and CGP conditions, and Fleiss’ κ was used to assess agreement across the three runs. In addition, Chinese reports were translated into English and re-entered into the model, and quadratic-weighted Cohen’s κ was used to assess agreement between outputs generated from Chinese and English inputs. All reported *p*-values were two-sided, and *p* < 0.05 was considered statistically significant; prespecified primary comparisons in the reader study used Holm correction to control for multiple comparison errors at the vessel and patient levels, respectively.

## Results

### Baseline cohort characteristics

The final study cohort comprised 433 patients (mean age, 65.9 years ± 13.4 [SD]; 248 males, 185 females), totaling 866 evaluable carotid arteries (Fig. 1). The study encompassed a range of disease severities (detailed in Table 1).

**Table 1:** Baseline characteristics and plaque classification distributions in the full cohort and study subsets

	Center A	Center B	Center C	CRFS study	Reader study
Total patients, *N*	376	28	29	150	200
Demographic and clinical characteristics					
Age, years, mean (SD)	66.1 (13.71)	63.44 (11.31)	66.52 (10.86)	56 (13.91)	55 (12.53)
Sex (male), *n* (%)	217 (57.71)	13 (46.43)	18 (62.07)	88 (58.67)	102 (51.00)
BMI, mean (SD)	24.43 (2.49)	24.39 (2.54)	25.04 (3.09)	24.54 (2.31)	24.28 (2.53)
Hypertension, *n* (%)	243 (64.63)	21 (75.00)	20 (68.97)	89 (59.33)	122 (61.50)
Diabetes, *n* (%)	123 (32.71)	9 (32.14)	7 (24.13)	50 (33.33)	45 (22.50)
History of stroke, *n* (%)	102 (27.12)	6 (21.43)	9 (31.04)	32 (21.33)	43 (21.50)
Modified AHA classification (vessel level), *n* (%)					
Normal/I–II	241 (32.05)	8 (14.29)	21 (36.21)	89 (29.67)	120 (30.00)
III	23 (3.06)	4 (7.14)	1 (1.72)	9 (3.00)	9 (2.25)
IV–V	150 (19.95)	19 (33.93)	10 (17.24)	59 (19.67)	65 (16.25)
VI	221 (29.39)	25 (44.64)	19 (32.76)	88 (29.33)	133 (33.25)
VII	34 (4.52)	0 (0.00)	1 (1.72)	13 (4.33)	16 (4.00)
VIII	83 (11.04)	0 (0.00)	6 (10.34)	38 (12.67)	57 (14.25)

Unless otherwise indicated, continuous data are presented as mean (SD) and categorical data as *n* (%). Subsets for the CRFS and reader studies were drawn by stratified random sampling to ensure adequate representation of rare categories

*AHA* American Heart Association, *BMI* body mass index, *CRFS* Clinical Reasoning Fidelity Score, *SD* standard deviation

### Report-based reader study

**Fig. 3:**
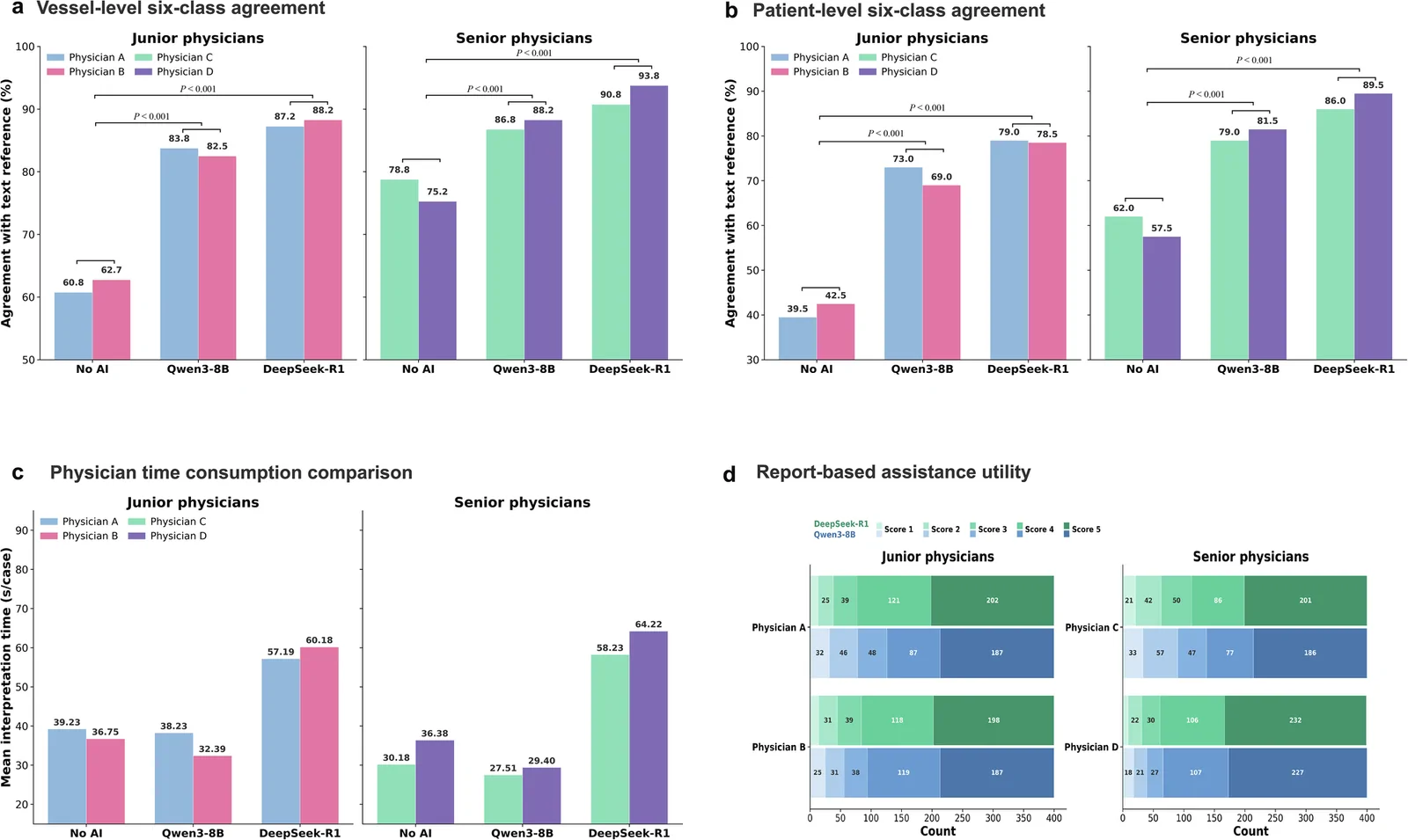
Reader study performance with AI assistance at the vessel and patient levels. **a** Vessel-level six-class agreement with the text-based reference standard for junior and senior radiologists under three reading conditions: no AI assistance, Qwen3-8B assistance, and DeepSeek-R1 assistance. **b** Patient-level six-class agreement with the text-based reference standard for junior and senior physicians under the same three reading conditions. **c** Mean interpretation time per case under the three reading conditions. **d** Distribution of Diagnostic Assistance Utility Score (DAUS) for Qwen3-8B and DeepSeek-R1; higher scores indicate greater perceived usefulness. Across both experience levels, AI assistance improved agreement with the text-based reference standard, with larger gains observed in junior radiologists, while Qwen3-8B was associated with shorter interpretation time than DeepSeek-R1

Artificial intelligence (AI) assistance improved agreement of report-based classification results with the text-based reference standard (Table 2 and Supplementary Table S12). In the vessel-level primary analysis, mean agreement without AI was 61.75% for junior and 77.00% for senior radiologists (Fig. 3a); with Qwen3-8B assistance, it increased to 83.12% and 87.50%, respectively, and with DeepSeek-R1 assistance to 87.75% and 92.25%. Compared with no AI, Qwen3-8B significantly improved agreement in both groups, and DeepSeek-R1 yielded additional gains over Qwen3-8B; all group-level confirmatory comparisons remained significant after Holm correction (all *p* < 0.001). Patient-level analyses were directionally consistent: agreement increased from 41.0% to 71.0% and 78.8% in junior radiologists and from 59.8% to 80.2% and 87.8% in senior radiologists (Fig. 3b; all Holm-corrected *p* < 0.001). Mean agreement with the reference standard (quadratic-weighted κ) increased from 0.041 to 0.603 and 0.714 in junior radiologists and from 0.421 to 0.722 and 0.836 in senior radiologists across the three conditions (Table 3). AI assistance also narrowed the experience gap: at the vessel level, the junior–senior agreement gap decreased from 15.25 percentage points without AI to 4.38 with Qwen3-8B and 4.50 with DeepSeek-R1, and the weighted κ gap decreased from 0.380 to 0.119 and 0.122, respectively. At baseline, senior radiologists outperformed junior radiologists at both vessel and patient levels (Supplementary Table S12, all Holm-corrected *p* < 0.001). Individual reader-level results were directionally consistent with the pooled analysis: all four readers showed improved six-class agreement with the text-based reference standard after AI assistance. Across individual readers, vessel-level agreement increased from 60.75–78.75% without AI to 82.50–88.25% with Qwen3-8B and 87.25–93.75% with DeepSeek-R1. Within-experience-group variation remained small across conditions (junior readers, 1.0–2.0 percentage points; senior readers, 1.5–3.5 percentage points). Detailed individual-level data are provided in Supplementary Tables S7A and S8 and Fig. S5. For Type VI detection, junior sensitivity increased from 60.9% without AI to 83.8% with Qwen3-8B and 88.7% with DeepSeek-R1, approaching senior performance (Table 3 and Fig. S5). In the Center A–only sensitivity analysis excluding cases from external centers, the main findings were unchanged: vessel-level agreement increased from 62.37% to 83.78% with Qwen3-8B assistance and 87.50% with DeepSeek-R1 assistance among junior readers, and from 77.53% to 88.70% and 92.95%, respectively, among senior readers. All paired AI-assisted comparisons remained significant (*p* < 0.001; Table S7B). Mean interpretation time with Qwen3-8B assistance (31.9 s/case) was 10.4% shorter than without AI (35.6 s/case), whereas DeepSeek-R1 increased to 60.0 s/case because of model inference latency (Fig. 3c and Table S8). Median physician ratings for AI assistance utility were 4–5 (Fig. 3d). Post hoc analysis of AI-assisted session order showed that DeepSeek-R1 remained superior to Qwen3-8B (90.00% vs 85.31%, *p* < 0.001), while the session effect was small and not significant (88.12% vs 87.19%; difference, 0.93 percentage points; *p* = 0.325), and the model difference was not significantly affected by AI-assisted reading order (*p* = 0.437).

**Table 2:** Reader study agreement with text-based reference standard

Group	Condition	Agreement (%)	Macro F1	Weighted κ
Junior (*n* = 2)	No AI	61.75 ± 1.00	0.460 ± 0.005	0.041 ± 0.015
	Qwen3-8B	83.12 ± 0.63	0.757 ± 0.010	0.603 ± 0.055
	DeepSeek-R1	87.75 ± 0.50	0.814 ± 0.013	0.714 ± 0.001
Senior (*n* = 2)	No AI	77.00 ± 1.75	0.685 ± 0.088	0.421 ± 0.192
	Qwen3-8B	87.50 ± 0.75	0.834 ± 0.032	0.722 ± 0.018
	DeepSeek-R1	92.25 ± 1.50	0.895 ± 0.028	0.836 ± 0.025
Overall (*n* = 4)	No AI	69.38 ± 7.76	0.572 ± 0.128	0.231 ± 0.234
	Qwen3-8B	85.31 ± 2.29	0.796 ± 0.046	0.663 ± 0.072
	DeepSeek-R1	90.00 ± 2.51	0.855 ± 0.046	0.775 ± 0.064

Data are mean ± SD across physicians within each group. Accuracy denotes six-class overall accuracy for the modified AHA classification at the vessel level (200 patients, 400 vessels). Junior radiologists had 4–5 years of experience; senior radiologists had 7–9 years of experience; none had dedicated neurovascular imaging subspecialty training

*AI* artificial intelligence, *F1* harmonic mean of precision and recall, *κ* quadratic-weighted Cohen’s kappa, *SD* standard deviation

**Table 3:** Experience-related differences in agreement with the text-based reference standard

Metric	No AI (baseline)	Qwen3-8B assistance	DeepSeek-R1 assistance
Agreement gap (pp)	15.25	4.38	4.50
Junior agreement (%)	61.75	83.12	87.75
Senior agreement (%)	77.00	87.50	92.25
Complex plaque (Type VI) sensitivity gap (pp)	9.80	−0.30	0.00
Junior sensitivity (%)	60.9	83.8	88.7
Senior sensitivity (%)	70.7	83.5	88.7
Agreement with reference standard (Weighted κ) gap	0.380	0.119	0.122
Junior κ	0.041	0.603	0.714
Senior κ	0.421	0.722	0.836

Gap values represent the difference in performance metrics between senior and junior radiologists (senior minus junior). All AI assistance conditions reduced the gap across all metrics. Under DeepSeek-R1 assistance, the Type VI sensitivity gap was reduced to 0.00 pp. Indented rows show the individual group values from which the gap was calculated

*AI* artificial intelligence, *κ* quadratic-weighted Cohen’s kappa, *pp* percentage points

**Fig. 4:**
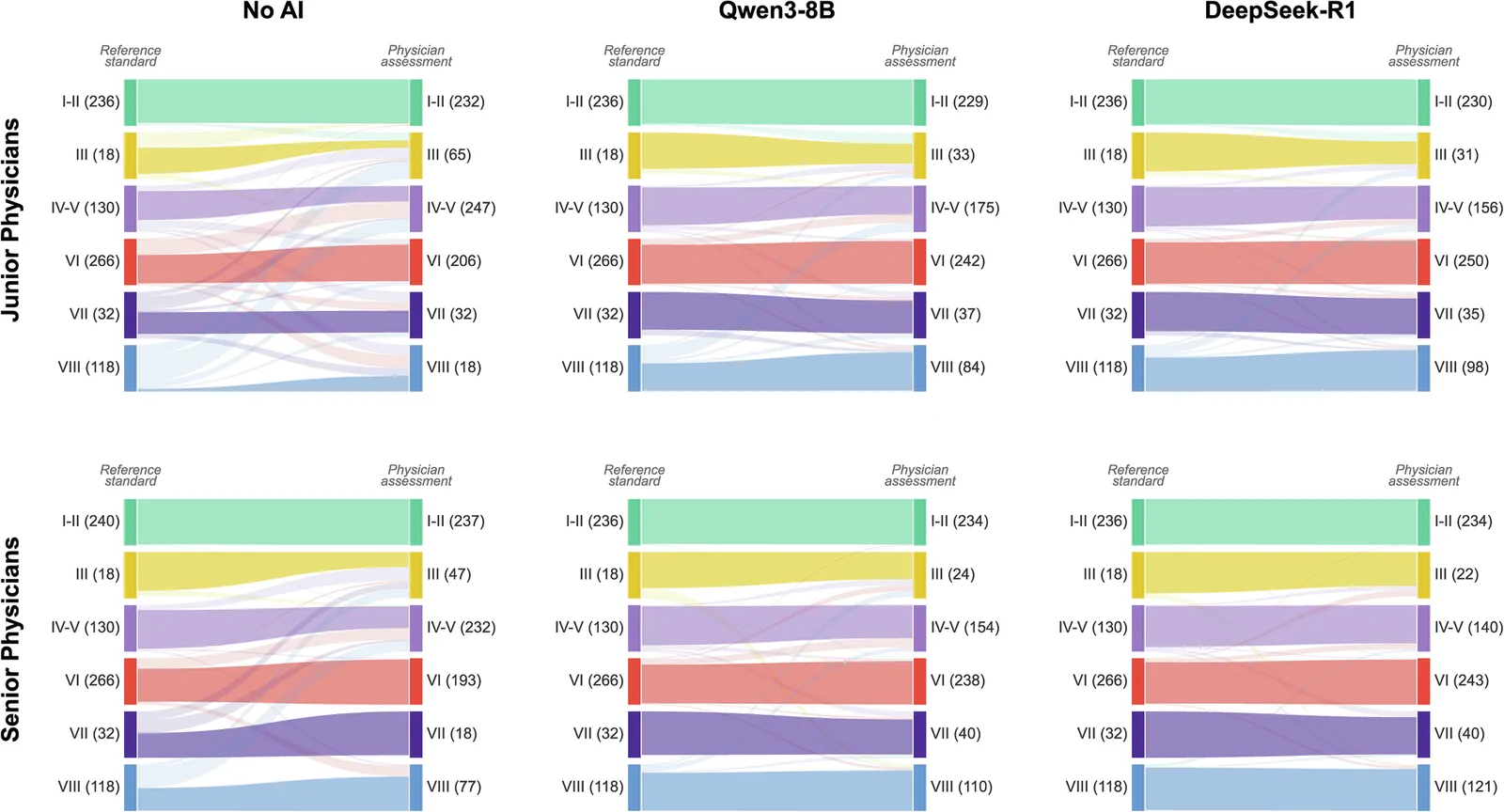
Sankey diagram of classification patterns in the human–AI collaboration study. Each panel displays the flow from reference-standard modified AHA types (left) to physician-assigned types (right). Rows represent physician experience levels (Junior: top; Senior: bottom), and columns represent assistance conditions (No AI, Qwen3-8B, DeepSeek-R1). The dominant misclassification pattern under unassisted conditions was Type VI being misclassified as Type IV–V (33.1% for junior physicians, 21.8% for senior physicians). AI assistance substantially reduced these misclassification flows, with DeepSeek-R1 achieving the greatest reduction (decreased by 76.1% for junior and 69.0% for senior physicians)

Misclassification analysis (Fig. 4) showed that, in the unaided condition, the predominant error was misclassifying type VI plaques as type IV–V, with 88 vessel-level errors among junior physicians and 58 among senior physicians. With DeepSeek-R1 assistance, this error among junior physicians decreased to 21, representing a 76.1% reduction. However, signals of automation bias remained. Among 800 vessel-level readings by junior physicians, 33 readings (4.1%) were correct without AI but became incorrect with AI assistance; a similar pattern was also observed among senior physicians. These errors were mainly concentrated in transitional boundary categories, most commonly type IV–V misclassified as type VI(12/33), type I–II misclassified as type III (6/33), and type VI misclassified as type IV–V (4/33). These errors typically occurred in cases with ambiguous report wording, overlapping plaque signal characteristics, or indistinct classification boundaries.

### Standalone AI performance evaluation

**Fig. 5:**
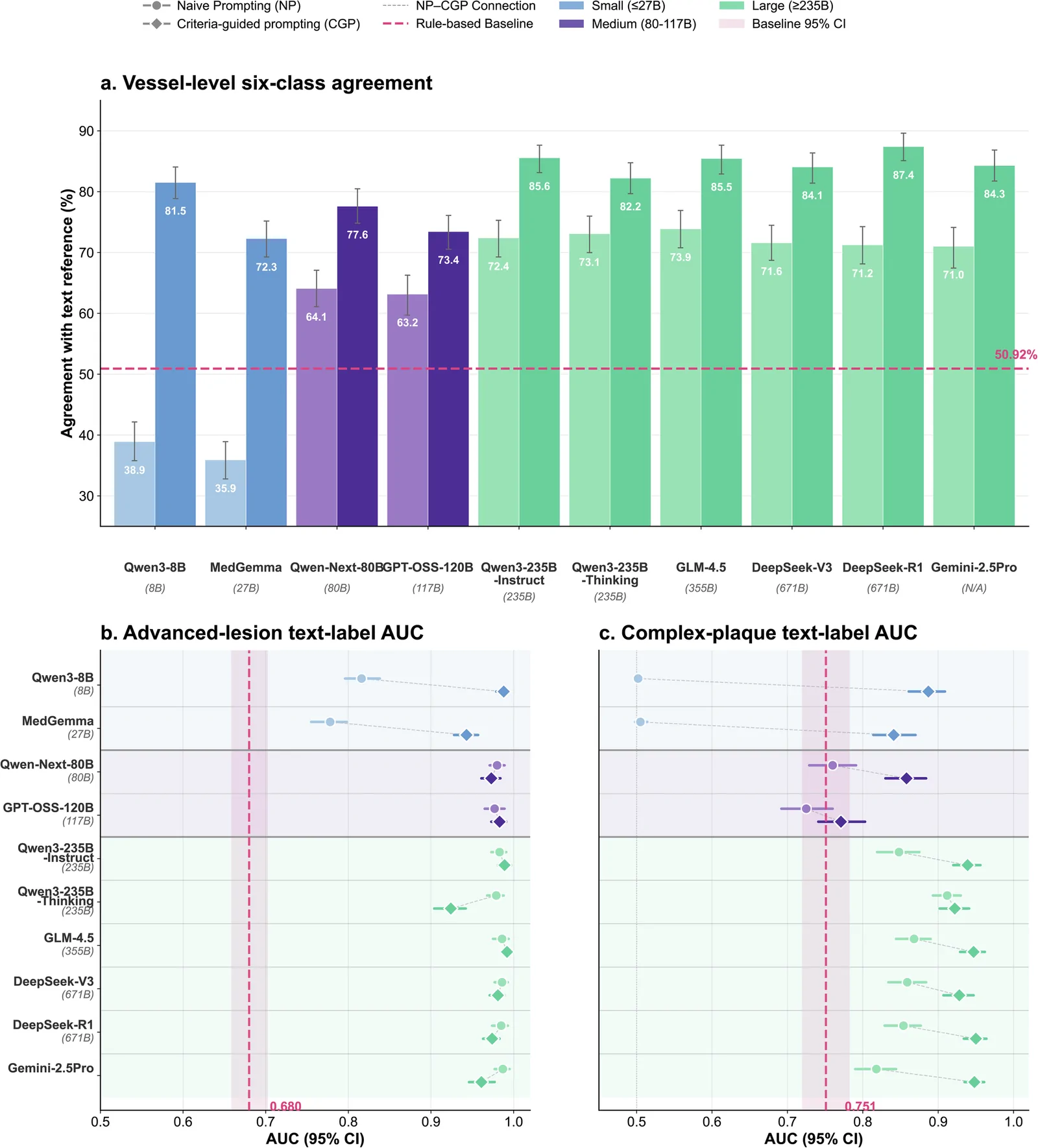
Classification performance of rule-based method and large language models. **a** Vessel-level six-class agreement with the text-based reference standard for the rule-based method and 10 large language models under Naive Prompting (NP) and criteria-guided prompting (CGP) conditions. Error bars represent 95% bootstrap confidence intervals. **b** Binary classification performance for advanced-lesion text-label classification (Normal/Type I–II versus Type III–VIII). Forest plot displaying area under the receiver operating characteristic curve (AUC) with 95% confidence intervals. **c** Binary classification performance for Type VI complex-plaque text-label classification (Type VI versus non-Type VI). Forest plot displaying AUC with 95% confidence intervals

The rule-based method achieved 50.92% six-class agreement. Under naive prompting (NP), most small models performed poorly (e.g., Qwen3-8B: 38.91%). When the prompt explicitly encoded the AHA criteria (CGP), all models showed higher agreement (Fig. 5a). The lightweight Qwen3-8B improved by 42.6 percentage points to 81.52% (95% CI: 78.87–84.06%), remaining 5.89 percentage points below DeepSeek-R1 (87.41%, 95% CI: 85.10–89.61%). The commercial closed-source Gemini 2.5 Pro achieved 84.30% under CGP, comparable to several open-source models (Table S4). Agreement varied across centers (Center A: 73–88%; Center B: 52–73%; Center C: 74–95%), and CGP reduced but did not eliminate this heterogeneity. Using Qwen3-8B as an example, three-center agreement under CGP was 82.05%, 69.64%, and 86.21%, respectively; the corresponding values for DeepSeek-R1 were 87.90%, 73.21%, and 94.83% (Supplementary Table S4). Because Centers B and C had small sample sizes (56 and 58 vessels, respectively), these estimates were descriptive only. The class distributions by center are provided in Supplementary Table S3. Repeated runs of Qwen3-8B under CGP showed high output stability, with higher agreement than NP (Fleiss’ κ: 0.91 vs 0.85). In the exploratory Chinese–English bilingual analysis, cross-language agreement was also higher under CGP than under NP (Cohen’s κ: 0.86 vs 0.73), as detailed in Supplementary Table S15. For regrouped binary tasks, AUCs were high for advanced lesion detection and more variable for Type VI detection [6, 24] (Fig. 5b, c). DeepSeek-R1 achieved the highest AUC for Type VI detection (0.950), whereas Qwen3-8B sensitivity for Type VI improved from 0.4% under NP to 80.1% under CGP. With CGP, more than 60% of reasoning outputs achieved a CRFS score of ≥ 4 (Fig. S4). In Qwen3-8B, CGP reduced low-scoring outputs (score ≤ 2) from 43.6% to 17.6% and increased higher-scoring outputs (score ≥ 4) from 34.5% to 72.0% (Table S6).

## Discussion

**Fig. 6:**
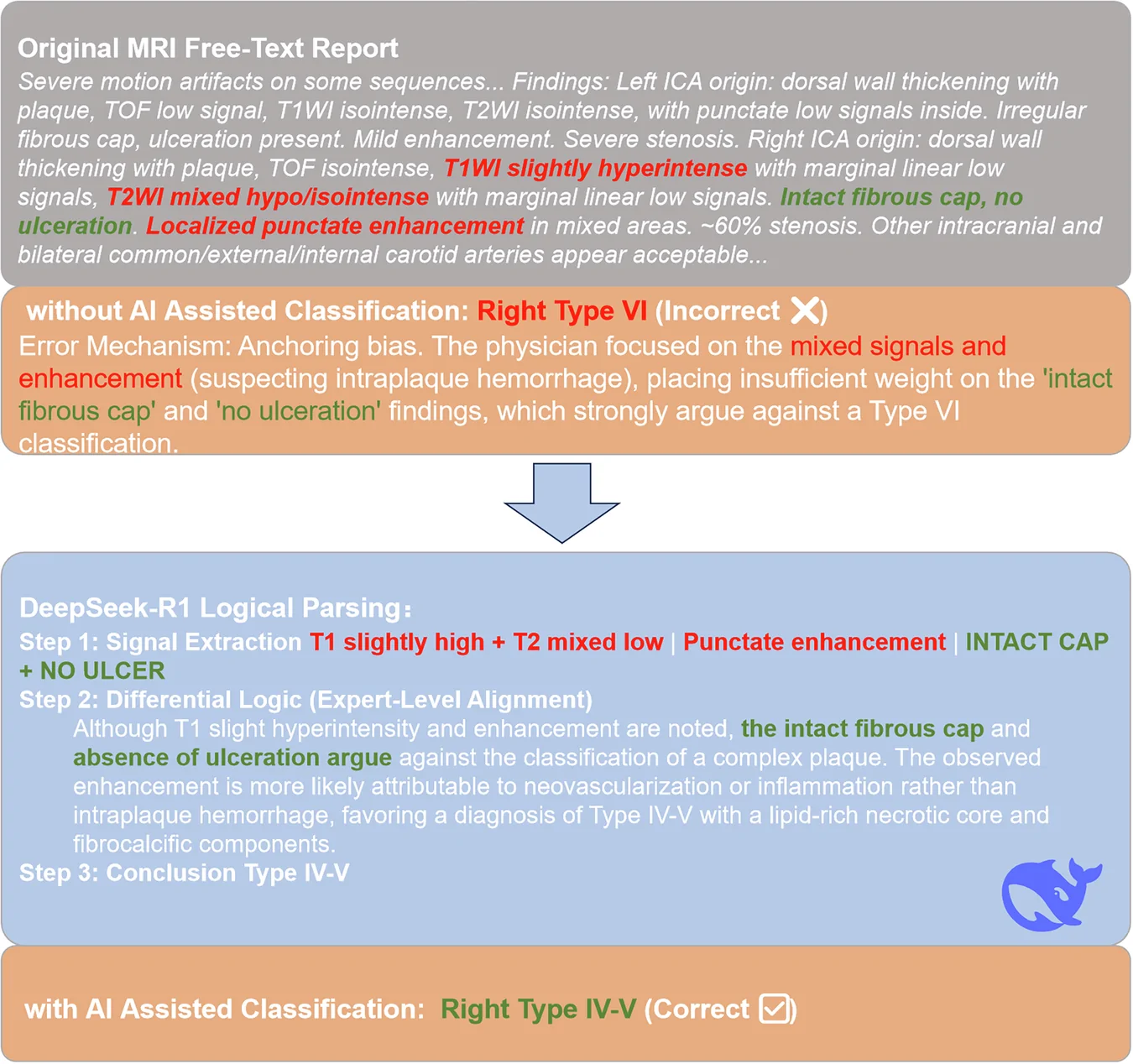
Representative case demonstrating AI preventing clinical overdiagnosis. A free-text carotid HRMRI report described bilateral plaques with motion artefacts on some sequences. A junior radiologist initially classified the right internal carotid artery plaque as Type VI. DeepSeek-R1 re-evaluated the reported imaging features, including slight T1 hyperintensity, mixed low/isointense T2 signal, focal enhancement, an intact fibrous cap, and absence of ulceration, and supported reclassification to Type IV–V. This example illustrates how auditable AI reasoning may help correct false-positive high-risk assignments

In this report-based reader study, large language model assistance improved six-class classification agreement with the text-based reference standard, with greater gains observed in junior radiologists. At the vessel level, the junior–senior agreement gap decreased from 15.25 percentage points without AI to 4.38 with Qwen3-8B and 4.50 with DeepSeek-R1. In standalone evaluation, explicit encoding of the AHA criteria in the prompt raised Qwen3-8B agreement from 38.91% to 81.52%, leaving a residual 5.89-percentage-point gap to DeepSeek-R1 (87.41%). Taken together, these findings support explicit criterion encoding as a practical strategy for standardizing report-based classification and enabling smaller local models (Fig. 6).

The error pattern analysis helps explain where this benefit arose. Without AI assistance, the dominant error was classifying Type VI plaques as Type IV–V, a clinically important distinction because Type VI plaques generally prompt closer evaluation than Type IV–V plaques [4, 8]. AI assistance, by providing a structured reasoning framework, may help transform physicians’ reliance on tacit clinical experience into a more explicit and reproducible logical process. In essence, it shifts classification from a predominantly experience-dependent task toward a description-driven collaborative process, which may partly explain the substantial reduction in missed Type VI lesions (76.1%) and the narrowing of the experience gap between junior and senior readers [25, 26]. However, AI assistance also introduced errors in borderline cases, such as between types IV–V and VI and between types I–II and III, indicating that model outputs should not be treated as automated decisions. Future implementation should include interface-level warnings that AI outputs are reference suggestions only and that final classification requires independent physician judgment, together with safeguards such as physician override, focused review of borderline outputs, and regular auditing of AI-discordant cases. System use should be restricted to report-based standardization assistance rather than autonomous diagnosis. Any future deployment should also be accompanied by local governance and continuous monitoring, including periodic audits of AI-assisted reports and AI–physician discordant cases, in line with multi-society guidance on radiology AI implementation and the FUTURE-AI framework for trustworthy healthcare AI [27, 28].

Our findings extend prior work showing that large language models can derive standardized radiologic categories from free-text reports. Matute-González et al [29]. demonstrated the feasibility of this text-to-classification paradigm for LI-RADS categorization, but their study was smaller, single-center, and did not quantify the incremental effect of AI assistance on physicians using a crossover design. This study extends the conceptual framework of our previously published work on diagnostic impression generation from brain MRI reports [14], expanding report-based large language model applications from diagnostic impression generation to modified AHA classification. The present cohort was independent from that reported in Reference 14, with no patient-level overlap. The latter requires integration of multi-sequence MRI signal characteristics and application of hierarchical exclusion criteria, representing a higher level of cognitive integration. In our data, explicit criteria encoding, not model scale alone, was a major determinant of performance. The observation that Qwen3-8B approached much larger models when the prompt encoded the modified AHA criteria increases the appeal of smaller models as practical candidates. Meanwhile, performance varied substantially across centers; however, because Centers B and C had limited sample sizes (56 and 58 vessels, respectively), these differences may partly reflect estimation instability due to small samples. Therefore, the cross-center applicability of this approach requires further validation in larger external cohorts. In the exploratory bilingual analysis, CGP also showed higher Chinese–English agreement than NP (Cohen’s κ, 0.86 vs 0.73; Table S15), suggesting a degree of robustness to language variation, although external validation in non-Chinese reports remains necessary. The 3.7-s per-case reduction with Qwen3-8B was modest but may be meaningful in high-throughput workflows, whereas DeepSeek-R1 increased reading time from 35.6 to 60.0 s per case, possibly due to slower generation and longer reasoning outputs requiring physician review. In accordance with Bluethgen et al’s best-practice recommendations for radiology LLM studies and the CLAIM checklist [23, 30], we provided full prompts, criteria tables, model information, inference parameters, output stability testing, and report-level evaluation boundaries to enhance reproducibility and transparency. A recent systematic review of 246 LLM studies in radiology found that model version, temperature, and full prompts were reported in fewer than half of the studies [31]; to support reproducibility, we reported all model specifications and inference parameters (Supplementary Methods S2 and S5).

This study also has important limitations. First, the reference standard was based on text-derived expert consensus, and the reader study was restricted to a report-only setting. Therefore, the findings reflect text-to-label mapping performance and are constrained by the quality and completeness of the original reports; they should not be interpreted as evidence of image-level diagnostic accuracy or downstream clinical improvement. Second, the reader design was not fully randomized: the unaided condition was always performed first to avoid contamination of baseline readings by AI exposure, which may have led learning effects to overestimate and fatigue effects to underestimate the benefit of AI assistance. In addition, bilateral vessels and repeated assessments by the same readers across multiple conditions meant that observations were not fully independent. Although post hoc session-order analysis showed no significant effect, residual bias cannot be excluded. Third, all original reports were in Chinese, and the cohort was dominated by Center A (376/433 patients), with limited external validation samples. Although preliminary Chinese–English analysis showed high cross-language agreement under CGP (Cohen’s κ = 0.86), this analysis involved translated reports rather than independently authored non-Chinese reports; therefore, the cross-language and cross-institutional generalizability of the approach requires larger-scale external validation. Fourth, no a priori sample size calculation was performed, but post hoc sensitivity analysis showed that the observed effect sizes for all confirmatory comparisons exceeded the minimum detectable thresholds (Supplementary Table S16). Moreover, only four readers were included, with two readers in each experience group, which limited the statistical robustness of comparisons between experience levels. The differences between junior and senior readers reported in the main text should therefore be interpreted as observed reader-level patterns. Finally, formal CRFS scoring was performed by a single evaluator, which may have introduced residual subjective bias. These limitations support the need for prospective, multi-reader, multi-case validation using image-level endpoints before broader clinical implementation.

## Conclusion

This study demonstrates that, in a report-based classification setting, LLM assistance can improve radiologists’ agreement with a text-based reference standard for carotid plaque AHA classification, with larger gains in junior radiologists. Explicit encoding of established AHA criteria in the prompt enabled an 8B model to achieve useful performance in this setting, supporting further evaluation of locally deployable tools for standardizing text-based classification workflows.

## Supplementary information

ELECTRONIC SUPPLEMENTARY MATERIAL

## Data Availability

The datasets generated and/or analyzed during the current study are not publicly available due to institutional and patient-privacy restrictions but are available from the corresponding author on reasonable request and subject to approval by the relevant institutions.

## Abbreviations

AHA: American Heart Association
AI: Artificial intelligence
AUC: Area under the receiver operating characteristic curve
CGP: Criteria-guided prompting
CI: Confidence interval
CLAIM: Checklist for Artificial Intelligence in Medical Imaging
CRFS: Clinical Reasoning Fidelity Score
HRMRI: High-resolution magnetic resonance imaging
LLM: Large language model
NP: Naive prompting

## Code availability

The code used in this study is publicly available in an online repository: https://github.com/Becomingw/CGP-Plaque.
