# Supplementary material for: Large language model assistance for report-based carotid plaque AHA classification: a multi-reader study
Source: Insights Imaging. 2026 Oct 16;17:264. doi: 10.1186/s13244-026-02421-2 (PMC13675435; doi:10.1186/s13244-026-02421-2)
Supplement: Supplementary file 1 [file 13244_2026_2421_MOESM1_ESM.pdf]

Large Language Model Assistance for Report-Based Carotid Plaque AHA Classification: A Multi-Reader Study

ELECTRONIC SUPPLEMENTARY MATERIALS

Supplementary Methods

S1. Rule-Based Baseline: Complete Specification

The rule-based classification system employed a three-layer decision architecture designed to mirror the cognitive process of radiologists interpreting HRMRI reports.

Layer 1: Direct Label Recognition

The system first scanned the report text for explicit classification labels using pattern matching:

- Patterns: "Type VI", "Type IV", "AHA VI", "AHA type VI", etc.
- If a direct label was detected, the system output that classification immediately without further processing.

Layer 2: Signal-Feature Extraction

If no direct label was present, the system extracted key imaging findings using predefined signal-to-component mapping rules:

Component	Detection Rules
Intraplaque Hemorrhage (IPH)	T1WI hyperintensity combined with TOF hyperintensity
Ulceration/Fibrous Cap Rupture	Keyword matching: "ulcer," "rupture," "surface defect," "fibrous cap discontinuity"
Lipid-Rich Necrotic Core (LRNC)	T2WI hypointensity without enhancement
Fibrous Tissue	Marked enhancement on contrast-enhanced imaging
Calcification	T1WI and T2WI hypointensity, signal void

Negation Detection: The system applied clause-level negation detection to reduce false positives. Negation phrases included: "not seen," "no evidence of," "without," "absent," "negative for," "ruled out."

Layer 3: Type Assignment

Based on extracted components, the system assigned types according to modified AHA classification criteria in the following priority order:

1. IPH or ulceration/fibrous cap rupture present → Type VI
2. Prominent calcification without lipid core → Type VII
3. Fibrous tissue without lipid core → Type VIII
4. Lipid core with surrounding fibrous tissue → Type IV-V
5. Fibrous tissue alone (intimal thickening) → Type III
6. None of the above → Type I-II (normal or near-normal)

The system output the final type along with supporting textual evidence from the report.

S2. Model Deployment and Inference: Technical Details

Hardware Configuration

All experiments were conducted on a local high-performance computing cluster equipped with:

- 8× NVIDIA H20 GPUs, each with 141 GB HBM3 memory
- 2× Intel Xeon Platinum 8480+ processors
- 2 TB DDR5 RAM
- Ubuntu 22.04 LTS operating system

Software Framework

- vLLM v0.8.4 for model deployment within Docker containers
- GPT-OSS-120B was executed with vLLM v0.10.1-oss due to compatibility requirements
- Python 3.12 with OpenAI-compatible API interface

Inference Parameters

Parameter	Value	Rationale
Temperature	0.1	Minimize output randomness for reproducible classification
max_tokens	8,192	Maximum output length to ensure complete reasoning chains
top_p	1.0 (default)	Standard sampling
Batch size	16	Optimized for throughput while maintaining memory constraints

Model-Specific Configurations

- Qwen3-8B: Built-in thinking mode was disabled based on pilot experiments showing substantially increased inference time and output-format noncompliance with thinking mode enabled.
- Gemini 2.5 Pro: Accessed via OpenRouter API (https://openrouter.ai; accessed August 12, 2025) with identical parameter settings.
- All other models: Used default inference modes as provided by developers.

Output Validation and Retry Logic

1. The system automatically validated JSON output format against expected schema.
2. Upon parsing failure, the system retried with the same prompt (maximum 3 attempts).
3. For samples that remained unparseable after 3 attempts, a junior physician (blinded to reference standard and model identity) manually extracted classifications from raw output to avoid introducing bias.
4. JSON parsing success rates ranged from 95.2% to 99.8% across models (Figure S1).

Reporting Transparency for Radiology LLM Evaluation

- Following the best-practice recommendations for large language models in radiology by Bluethgen et al. and the CLAIM checklist, we reported the full prompts and criteria tables (Supplementary Methods S5), model names, scales, language support, release/version dates, and deployment routes (Table S2), inference parameters and retry logic (Supplementary Methods S2), output-stability testing (Table S15), and the report-based text-to-label evaluation boundary. These details are intended to support reproducibility and clarify that the evaluation was performed at the report-text level rather than as an image-level diagnostic accuracy study.

S3. Clinical Validation Study: Detailed Design

Study Design Overview

- The human-AI comparison study employed a fixed-order unassisted-first design with counterbalanced AI-assistance sequence to evaluate report-based agreement with the text-based reference standard while reducing, but not eliminating, learning and period effects.

Participant Selection and Training

- Four radiologists from Shanghai Sixth People's Hospital participated:
  - 2 junior physicians (4–5 years of carotid HRMRI interpretation experience)
  - 2 senior physicians (7–9 years of experience)
- Prior to the study, all physicians received standardized training covering:
  - Modified AHA classification criteria and decision rules
  - Web-based system operation and interface navigation
  - Practice cases (n=10, excluded from analysis)
- Physicians were not informed of model performance characteristics or the study's hypotheses regarding experience-related differences.

Experimental Phases

Phase	Duration	Condition	Case Order
Phase 1	2 weeks	No AI assistance (baseline)	Randomized per physician
Washout	6 weeks	—	—
Phase 2	2 weeks	AI Model A (randomized assignment)	Re-randomized
Washout	6 weeks	—	—
Phase 3	2 weeks	AI Model B (counterbalanced)	Re-randomized

Randomization and Counterbalancing

- Physicians A and C received Qwen3-8B in Phase 2, DeepSeek-R1 in Phase 3.
- Physicians B and D received DeepSeek-R1 in Phase 2, Qwen3-8B in Phase 3.
- Case presentation order was independently randomized for each phase using a computer-generated random sequence (seed fixed per physician-phase combination for reproducibility).
- 6-week washout periods between phases were used to reduce memory effects from repeated case exposure.

Web-Based Assessment System

The custom-developed platform (Figure S2) provided:

- Display of de-identified free-text HRMRI reports
- Dropdown menus for left and right carotid classification selection
- Under AI-assisted conditions: expandable panel showing LLM-suggested classifications with complete reasoning chains
- Interface-level warning that AI outputs were reference suggestions only and that final classification required independent physician judgment
- Automatic timestamping of case entry and submission for time analysis
- Post-submission DAUS rating interface (displayed after each case under AI conditions)

Interpretation Time Measurement

- Defined as the duration from entering the case page to submitting the classification
- Under AI assistance, this included both:
    - Model inference latency (time from page load to AI result display)
    - Physician decision time (time from AI result display to submission)
- DAUS rating time was excluded from interpretation time calculations

Diagnostic Assistance Utility Score (DAUS)

Physicians rated AI usefulness on a 1–5 scale separately for each carotid side:

- 5: AI output substantially increased decision confidence or corrected my initial tendency
- 4: AI output was helpful and aligned with my interpretation
- 3: AI output was partially useful but required careful evaluation
- 2: AI output was minimally helpful
- 1: AI output was not helpful or was misleading

Blinding

- Physicians remained double-blinded throughout:
    - Blinded to reference-standard labels until study completion
    - Blinded to model identity (AI outputs were presented without model names)
- Unblinding occurred only after all data collection was complete

S4. Statistical Analysis: Complete Methods

Bootstrap Confidence Interval Estimation

- All confidence intervals for report-based agreement metrics were estimated using bootstrap resampling.
- Parameters: 1,000 bootstrap samples, random seed fixed at 42 for reproducibility.
- Percentile method (2.5th and 97.5th percentiles) was used for interval construction.

Rationale for Macro F1 over Weighted F1

We reported macro F1 rather than weighted F1 as the primary classification metric for two reasons:

1. Clinical significance of rare types: Low-frequency types (Type III: 3.2%; Type VII: 4.0%) represent specific stages of plaque evolution with distinct clinical implications. Weighted averaging would dilute performance assessment for these clinically important categories.
2. Sensitivity to minority-class recognition: Macro averaging assigns equal weight to each class, making it more sensitive to minority-class recognition failure. This prevents overall performance from being dominated by high-frequency classes (Normal/I-II: 31.2%; Type VI: 30.6%).

Weighted F1 is additionally reported in Table S14 for completeness.

Agreement Evaluation (Cohen's κ)

- Quadratic-weighted Cohen's κ was used to account for the ordinal nature of the classification (severity increases from Type I-II to Type VI).
- Interpretation followed Landis and Koch criteria:
  - <0.20: Poor agreement
  - 0.21–0.40: Fair agreement
  - 0.41–0.60: Moderate agreement
  - 0.61–0.80: Substantial agreement
  - 0.80: Almost perfect agreement

McNemar's Test Implementation

- For standalone model comparisons, McNemar's test with Yates' continuity correction was used for paired agreement comparisons.
- Formula: $\chi^2 = (|b - c| - 1)^2 / (b + c)$, where b and c are discordant pairs.
- Reader-specific paired comparisons in the human-AI study used exact McNemar tests, implemented as two-sided exact binomial tests on discordant pairs.

Post Hoc Sensitivity Analysis for Reader-Study Sample Size

- Because no formal a priori sample size calculation was performed, we conducted a post hoc sensitivity analysis to clarify the effect-size range detectable by the available paired reader-study sample.
- Under a two-sided exact McNemar/binomial framework with 80% power and $\alpha = 0.05$, the observed discordance ranges corresponded to minimum detectable differences in agreement of approximately 6-8 percentage points at the vessel level and 11-14 percentage points at the patient level.
- The observed reader-specific No AI vs Qwen3-8B improvements exceeded these thresholds: 8.0-23.0 percentage points at the vessel level and 17.0-33.5 percentage points at the patient level.
- This post hoc analysis does not replace an a priori power calculation; smaller effects, such as 5 percentage points, may not have been reliably detectable. Because bilateral vessels from the same patient are correlated, vessel-level sensitivity estimates should be interpreted as approximate and descriptive.

Inter-Rater Reliability for CRFS

- Inter-rater reliability: ICC(2,1) (two-way random effects, single measures, absolute agreement)
- ICC interpretation per Koo and Li criteria:
    - <0.50: Poor
    - 0.50–0.75: Moderate
    - 0.75–0.90: Good
    - 0.90: Excellent

Multiple Comparison Correction

To control family-wise error rate (FWER) in multi-model and multi-condition comparisons, we applied Bonferroni correction within pre-specified confirmatory test families:

**Test Family**	**Number of Tests**	**Corrected α**
LLM vs Rule-based baseline (CGP)	10	Bonferroni
CGP vs NP (within model)	10	Bonferroni
Reader Study primary contrasts	3	Holm
Reader Study group contrasts, patient level	5	Holm

Reader-specific paired comparisons in the human-AI study were considered secondary analyses. Their p-values were Holm-adjusted within each endpoint/comparison family across the four physicians. Pairwise model matrices and CRFS/DAUS stratified analyses remained exploratory.

Statistical Software

- Python 3.12
- SciPy 1.11.4 for statistical tests (McNemar, Wilcoxon, ICC)
- scikit-learn 1.3.2 for classification metrics (agreement, F1, AUC)
- statsmodels 0.14.1 for confidence interval estimation

S5. Prompting Strategies

NP (Naive Prompting) Prompt

Role
Please act as a professional Medical Imaging Analysis Expert. Your task is to analyze the provided MRI imaging findings based on the American Heart Association (AHA) criteria for MRI plaque characterization and determine the corresponding AHA lesion type. Return the results in JSON format.

```
{
"Reasoning Process": "[Describe your analytical and reasoning steps step-by-step here]",
"AHA_Classification": {
  "Left": "[None OR the final analyzed AHA type (Roman Numerals)]",
  "Right": "[None OR the final analyzed AHA type (Roman Numerals)]"
}
}
```

Now, please analyze the following MRI imaging findings:

CGP (Knowledge-Guided Prompting) Prompt

Role
Please act as a professional Medical Imaging Analysis Expert. Your task is to analyze the provided MRI imaging findings based on the American Heart Association (AHA) criteria for MRI plaque characterization and determine the corresponding AHA lesion type. Return the results in JSON format.

AHA Plaque Classification Criteria
Type	Description
Type I-II	Near-normal wall thickening without calcification.
Type III	Diffuse intimal thickening or small eccentric non-calcified plaque (approx. 30% are mild stenosis).
Type IV-V	Plaque with a lipid or necrotic core surrounded by fibrous tissue; may contain calcification; no hemorrhage.
Type VI	Complex plaque with possible surface defects, hemorrhage (IPH), thrombus, or fibrous cap rupture.
Type VII	Calcified plaque.
Type VIII	Fibrous plaque without a lipid core; may contain small calcifications.

AHA Plaque Signal Characteristic Standards
Component	TOF	T1WI	T2WI	Enhancement
Lipid Core	Isointense	Iso/Hyperintense	Hypointense	No enhancement
Fibrous Tissue	Isointense	Iso/Hyperintense	Iso/Hyperintense	Enhancement (+)
Calcification	Low signal	Very low signal	Very low signal	No enhancement
Hemorrhage	High signal	High signal	Variable (High/Iso/Low)	No enhancement

Summary of Key Differential Points

Type	Lipid Core	T2WI Signal	Enhancement	Cap Integrity	Hemorrhage/Thrombus/Ulcer
Type IV-V	Present	Hypointense	Peripheral	Intact	None
Type VI	±	Variable	Peripheral	Ruptured/Defect/Irregular	Present
Type VIII	Absent	Isointense	Main body	Intact	None

Requirements

1. Thoroughly review and understand the AHA classification criteria mentioned above.
2. Separate the analysis for the **left and right carotid arteries**.
3. Correlate the signal descriptions in the imaging findings with the likely **pathological components** of the plaque.
4. Explain for each side which features match a specific AHA type. Rule out inapplicable types and provide justifications.
5. Explicitly state the final **AHA Classification** that best fits the MRI findings. For post-operative vessels or total occlusions, return "NA".
6. **Note:** Degree of stenosis is not a necessary condition for AHA plaque typing; the composition and structural features (e.g., lipid core, cap integrity, hemorrhage, calcification) are the decisive criteria.

> **Attention:** If plaques are present in both vessels, analyze each side separately.

```
{
"Reasoning Process": "[Describe your analytical and reasoning steps step-by-step here]",
"AHA_Classification": {
  "Left": "[None OR the final analyzed AHA type (Roman Numerals)]",
  "Right": "[None OR the final analyzed AHA type (Roman Numerals)]"
}
}
```

Now, please analyze the following MRI imaging findings:

Notes: NP provides minimal instructions requiring models to rely on internal knowledge. CGP includes comprehensive classification criteria, signal characteristics,

and differential diagnosis guidance to standardize the reasoning process. Please see details at: https://anonymous.4open.science/r/CGP-Plaque-68F2/Prompt/

S6. Illustrative Case: NP vs CGP Classification Comparison (Qwen3-8B)

Patient: 65-year-old male. **Reference standard:** Right AHA Type IV-V; Left AHA Type III.

HRMRI Report

Bilateral common carotid arteries and their internal and external carotid artery branches were visualized with slightly irregular vessel walls. Right internal carotid artery origin: plaque formation, showing TOF hypointensity, T1WI slight hyperintensity, T2WI iso-to-hyperintensity, with patchy hypointense areas within; fibrous cap was slightly irregular with an uneven surface; approximately 80–90% luminal stenosis. Left common carotid bifurcation and internal carotid artery origin: diffuse intimal thickening with small plaque formation, showing TOF isointensity, T1WI slight hyperintensity, T2WI iso-to-hyperintensity; approximately 30% luminal stenosis.

NP Output

Reasoning: Based on the AHA plaque classification criteria, the right internal carotid artery plaque shows TOF hypointensity (T1 hypointensity), T2WI iso-to-hyperintensity, with patchy hypointense areas internally (suggesting lipid core or fibrous cap incompleteness), and an irregular fibrous cap, consistent with Type B plaque (fibrous cap incomplete). The left carotid plaque shows diffuse thickening with small plaque, TOF isointensity (T1 isointensity), T2WI iso-to-hyperintensity, no obvious hypointense areas, and an intact fibrous cap, consistent with Type A plaque (fibrous cap intact). Degree of stenosis does not affect classification and serves only as supplementary information.

Classification: Left: A; Right: B

CGP Output

Reasoning: Analysis of the right internal carotid artery origin plaque: TOF hypointensity, T1WI slight hyperintensity, T2WI iso-to-hyperintensity with patchy hypointense areas suggest a possible lipid core (lipid core appears as TOF hypointensity and T2WI hypointensity). The slightly irregular fibrous cap with uneven surface suggests possible cap rupture or defect, consistent with Type VI characteristics (complex plaque with possible surface defect, hemorrhage, thrombus, or fibrous cap rupture). No definitive hemorrhage or thrombus signals were identified (acute hemorrhage would appear as TOF, T2WI hyperintensity), thus not supporting hemorrhage/thrombus features of Type VI. No calcification signals were identified, thus not supporting Type VII or Type VIII. Overall, the right-sided plaque conforms to Type VI. Analysis of the left common carotid bifurcation and internal carotid artery origin plaque: TOF isointensity, T1WI slight hyperintensity, T2WI iso-to-hyperintensity suggest no obvious lipid core. No calcification, hemorrhage, or fibrous cap defect identified. Diffuse intimal thickening with small plaque conforms to Type III (diffuse intimal thickening or small eccentric non-calcified plaque).

Classification: Left: III; Right: VI

Supplementary Tables

Table S1. MR Imaging Examination Acquisition Parameters

Sequence	Dataset	Manufacturer	Field Strength	Sequence Type	Echo Time (ms)	Flip Angle (°)	Slice Thickness (mm)	Pixel Spacing (mm)	Image Size
TOF-MRA	Development/ Internal test	Siemens	3T	GR	3.11	20	1.00	0.625	512×512×12
		Philips	3T	GR	3.45	20	1.20	0.391	512×512×168
		UIH	5T	GR	3.10	16	0.60	0.343	524×640×186
	External validation	GE	3T	GR	2.30	20	1.40	0.43	512×512×180
T1WI	Development/ Internal test	Siemens	3T	SE	18.00	120	0.88	0.438	544×80×640
		Philips	3T	SE	27.33	90	0.60	0.567	400×100×400
		UIH	5T	SE	14.94	79	0.40	0.399	376×451×552
	External validation	GE	3T	SE	27.94	80	3.00	0.156	512×512×10
T2WI	Development/ Internal test	Siemens	3T	SE	124.00	120	0.88	0.438	544×80×640
		Philips	3T	SE	229.79	90	1.00	0.463	432×180×432
		UIH	5T	SE	122.32	61	0.40	0.399	376×451×552
	External validation	GE	3T	SE	21.68	80	3.00	0.313	512×512×32
CE	Developme	Siemens	3T	SE	18.00	120	0.88	0.438	544×80×640

nt/<br>Internal test	Philips	3T	SE	27.33	90	0.60	0.567	400×100×400
	UIH	5T	SE	14.94	79	0.40	0.399	376×451×552
External validation	GE	3T	SE	24.86	80	3.00	0.156	512×512×10

Notes:

- TOF-MRA: Time-of-Flight Magnetic Resonance Angiography.
- T1WI: T1-weighted imaging.
- T2WI: T2-weighted imaging.
- CE: Contrast-enhanced imaging.
- GR: Gradient echo.
- SE: Spin echo.
- UIH: United Imaging Healthcare.

Table S2. LLM Information and VRAM Estimation

A. Detailed Information on the LLMs Used in This Study

Model	Model Size	Developer	Type	License	Language Support	Version Date	Reference
DeepSeek-R1	671B	DeepSeek-AI	General	MIT License	Chinese, English	2025.5	https://arxiv.org/abs/2501.12948
DeepSeek-V3	671B	DeepSeek-AI	General	MIT License	Chinese, English	2025.5	https://arxiv.org/abs/2501.12948
GLM-4.5	355B	Zhipu AI	General	MIT License	Chinese, English	2025.7	https://doi.org/10.48550/arXiv.2508.06471
Qwen3-235B-Thinking-2507	235B	Alibaba	General	Apache License 2.0	Chinese, English	2025.7	https://doi.org/10.48550/arXiv.2505.09388
Qwen3-235B-Instruct-2507	235B	Alibaba	General	Apache License 2.0	Chinese, English	2025.7	https://doi.org/10.48550/arXiv.2505.09388
Gemini 2.5 Pro	175B	Google	General	Proprietary (Google)	Chinese, English	2025.6	https://doi.org/10.48550/arXiv.2507.06261

GPT-OSS-120B	117B	OpenAI	General	Apache License 2.0	Chinese, English	2025.8	https://doi.org/10.48550/arXiv.2508.10925
Qwen3-Next-80B-A3B	80B	Alibaba	General	Apache License 2.0	Chinese, English	2025.9	https://doi.org/10.48550/arXiv.2505.09388
MedGemma 27B Instruct	27B	Google	Medical	Apache License 2.0	Chinese, English	2025.5	https://doi.org/10.48550/arXiv.2507.05201
Qwen3-8B	8B	Alibaba	General	Apache License 2.0	Chinese, English	2025.4	https://doi.org/10.48550/arXiv.2505.09388

Notes:

- Model sizes represent total parameters.
- MedGemma is the only medical domain-specific model; all others are general-purpose LLMs.

B. Native Precision and Single-Request 4k Context VRAM Estimates (March 2026)

Model	Native Precision	Total Parameters (Active Parameters)	Weight Memory (GB)	KV Cache ≈ (GB)	Total Estimated VRAM (incl. overhead)	Recommended GPU Setup (H100 80GB)	Performance Reference (decode tokens/s)
Qwen3-8B	BF16	8B	~16	~1	20–25 GB	1x (or even RTX 4090)	120–220
MedGemma (27B)	BF16	27B	~54	~2	60–70 GB	1x H100	80–150
Qwen-Next-80B-A3B	FP8	80B (3B)	~82	~2	95–115 GB	2x H100	90–170
GPT-OSS-120B	MXFP4	117B (5.1B)	~62–70	~2	80–100 GB	1x H100 (official target)	70–140
Qwen3-235B-Instruct / Thinking	FP8	235B (22B)	~235–250	~4–5	280–340 GB	4–5x H100 / 4x H200	50–120
GLM-4.5	FP8	355B (32B)	~355–370	~4	400–460 GB	6x H100 / 5x H200	40–100
DeepSeek-V3	FP8	671B (37B)	~685–700	~3–5 (MLA)	730–850 GB	9–11x H100 / 8x H200	45–110
DeepSeek-R1	FP8	671B (37B)	~685–700	~3–5	730–850 GB	Same as above	45–110

Notes:

- This table re-estimates VRAM using each model's officially supported native precision (not default BF16/FP16), assuming single concurrency and 4k context.
- Estimates are based on publicly available specifications as of March 2026,

using vLLM/SGLang documentation, and config-level verification.

- For additional estimation details, visit: https://apxml.com/zh/tools/vram-calculator

Table S3. Modified AHA Classification Distribution by Center (Evaluable Vessels)

Type	Center A (n=752)	Center B (n=56)	Center C (n=58)	Total (n=866)
Normal/I-II	241 (32.05%)	8 (14.29%)†	21 (36.21%)	270 (31.18%)
III	23 (3.06%)	4 (7.14%)†	1 (1.72%)†	28 (3.23%)
IV-V	150 (19.95%)	19 (33.93%)†	10 (17.24%)†	179 (20.67%)
VI	221 (29.39%)	25 (44.64%)	19 (32.76%)†	265 (30.60%)
VII	34 (4.52%)	0 (0.00%)†	1 (1.72%)†	35 (4.04%)
VIII	83 (11.04%)	0 (0.00%)†	6 (10.34%)†	89 (10.28%)

Notes:

- Values are n (%). † indicates n<20; performance estimates for these categories should be interpreted with caution due to wide confidence intervals.
- Center A: Shanghai Sixth People's Hospital.
- Center B: Tongji University Shanghai East Hospital.
- Center C: Huai'an First People's Hospital.

Table S4. Six-Class Classification Performance for All Models

	Condition	Six-class Agreement (95% CI)	Macro F1 (95% CI)	Center A	Center B	Center C
	–	50.92%	0.286	52.26%	23.21%	60.34%
	NP	38.91% (35.79–42.15)	0.182 (0.168–0.197)	39.49%	25.00%	44.83%
	CGP	81.52% (78.87–84.06)	0.717 (0.676–0.756)	82.05%	69.64%	86.21%
	NP	35.91% (32.79–38.92)	0.163 (0.146–0.178)	36.57%	21.43%	41.38%
	CGP	72.29% (69.28–75.18)	0.560 (0.516–0.603)	73.14%	58.93%	74.14%
	NP	64.09% (61.09–67.09)	0.435 (0.398–0.471)	64.63%	60.71%	60.34%
	CGP	77.60% (74.82–80.48)	0.671 (0.625–0.713)	79.52%	51.79%	77.59%

NP	63.16% (59.70–66.28)	0.500 (0.456–0.540)	63.83%	62.50%	55.17%
CGP	73.44% (70.55–76.10)	0.671 (0.625–0.708)	74.34%	60.71%	74.14%
NP	72.40% (69.28–75.29)	0.583 (0.535–0.627)	71.94%	78.57%	72.41%
CGP	85.57% (83.14–87.64)	0.774 (0.730–0.814)	86.44%	67.86%	91.38%
NP	73.09% (69.98–75.98)	0.485 (0.439–0.531)	73.14%	66.07%	79.31%
CGP	82.22% (79.68–84.76)	0.736 (0.698–0.770)	82.71%	66.07%	91.38%
NP	73.90% (70.78–76.91)	0.612 (0.570–0.652)	73.80%	78.57%	70.69%
CGP	85.45% (82.91–87.64)	0.767 (0.722–0.805)	86.97%	64.29%	86.21%
NP	71.59% (68.71–74.48)	0.496 (0.453–0.533)	71.01%	78.57%	72.41%

	CGP	84.06% (81.41–86.37)	0.765 (0.719–0.800)	85.24%	66.07%	86.21%
	NP	71.25% (68.13–74.25)	0.562 (0.527–0.599)	71.68%	64.29%	72.41%
	CGP	87.41% (85.10–89.61)	0.841 (0.804–0.871)	87.90%	73.21%	94.83%
	NP	71.02% (67.44–74.13)	0.567 (0.527–0.609)	71.94%	60.71%	68.97%
	CGP	84.30% (81.75–86.84)	0.770 (0.730–0.803)	85.37%	62.50%	91.38%

Notes:

- Best overall performance highlighted in bold.
- NP: Naive Prompting; CGP: Criteria-Guided Prompting.
- Center A: Shanghai Sixth People's Hospital; Center B: Tongji University Shanghai East Hospital; Center C: Huai'an First People's Hospital.
- 95% confidence intervals were calculated using bootstrap resampling (n=1000).

Table S5. Complete Binary Classification Performance Metrics

A. Advanced Lesion vs Non-advanced Lesion Detection

Model	Condition	Sensitivity	Specificity	PPV	NPV	AUC (95% CI)
Rule-based	—	37.4%	98.5%	98.2%	41.6%	0.680 (0.659–0.702)
Qwen3-8B	NP	64.8%	98.5%	99.0%	55.9%	0.816 (0.796–0.838)
Qwen3-8B	CGP	98.7%	98.9%	99.5%	97.1%	0.988 (0.979–0.995)
MedGemma	NP	57.4%	98.1%	98.6%	51.1%	0.778 (0.755–0.798)
MedGemma	CGP	90.4%	98.1%	99.1%	82.3%	0.943 (0.928–0.957)
Qwen-Next-80B	NP	96.8%	99.3%	99.7%	93.4%	0.980 (0.971–0.989)
Qwen-Next-80B	CGP	98.0%	96.7%	98.5%	95.6%	0.973 (0.961–0.984)
GPT-OSS-120B	NP	98.8%	96.7%	98.5%	97.4%	0.977 (0.965–0.989)
GPT-OSS-120B	CGP	97.7%	98.9%	99.5%	95.0%	0.983 (0.973–0.991)
Qwen3-235B-Instruct	NP	97.7%	98.9%	99.5%	95.0%	0.983 (0.973–0.991)
Qwen3-235B-Instruct	CGP	99.3%	98.5%	99.3%	98.5%	0.989 (0.981–0.997)
Qwen3-235B-Thinking	NP	97.3%	98.5%	99.3%	94.3%	0.979 (0.968–0.988)
Qwen3-235B-Thinking	CGP	93.6%	91.1%	95.9%	86.6%	0.924 (0.904–0.942)
GLM-4.5	NP	99.3%	97.8%	99.0%	98.5%	0.986 (0.975–0.994)
GLM-4.5	CGP	98.8%	99.6%	99.8%	97.5%	0.992 (0.986–0.997)
DeepSeek-V3	NP	98.3%	98.9%	99.5%	96.4%	0.986 (0.977–0.993)
DeepSeek-V3	CGP	97.7%	98.5%	99.3%	95.0%	0.981 (0.971–0.989)

DeepSeek-R1	NP	98.5%	98.5%	99.3%	96.7%	0.985 (0.974−0.993)
DeepSeek-R1	CGP	95.5%	99.3%	99.6%	90.8%	0.974 (0.963−0.983)
Gemini-2.5Pro	NP	98.8%	98.5%	99.3%	97.4%	0.987 (0.977−0.995)
Gemini-2.5Pro	CGP	99.3%	93.0%	96.9%	98.4%	0.961 (0.946−0.977)

B. Type VI (Complex Plaque) vs Non-VI Detection

Model	Condition	Sensitivity	Specificity	PPV	NPV	AUC (95% CI)
Rule-based	–	57.4%	92.8%	77.9%	83.2%	0.751 (0.720−0.782)
Qwen3-8B	NP	0.4%	100%	100%	69.5%	0.502 (0.500−0.506)
Qwen3-8B	CGP	80.1%	97.3%	93.0%	91.7%	0.887 (0.861−0.909)
MedGemma	NP	1.5%	99.5%	57.1%	69.5%	0.505 (0.498−0.514)
MedGemma	CGP	76.3%	91.8%	80.6%	89.7%	0.841 (0.814−0.870)
Qwen-Next-80B	NP	63.4%	88.7%	71.2%	84.6%	0.760 (0.729−0.791)
Qwen-Next-80B	CGP	78.1%	93.5%	84.1%	90.6%	0.858 (0.830−0.884)
GPT-OSS-120B	NP	53.6%	91.5%	73.6%	81.7%	0.725 (0.692−0.760)
GPT-OSS-120B	CGP	58.1%	96.2%	87.0%	83.9%	0.771 (0.741−0.803)
Qwen3-235B-Instruct	NP	79.6%	90.0%	77.9%	90.9%	0.848 (0.819−0.875)
Qwen3-235B-Instruct	CGP	93.2%	94.7%	88.5%	96.9%	0.939 (0.920−0.956)
Qwen3-235B-Thinking	NP	94.7%	87.7%	77.3%	97.4%	0.912 (0.893−0.930)

Qwen3-235B-Thinking	CGP	90.6%	93.8%	86.7%	95.7%	0.922 (0.902−0.941)
GLM-4.5	NP	88.3%	85.2%	72.5%	94.3%	0.868 (0.844−0.890)
GLM-4.5	CGP	93.6%	95.8%	90.9%	97.1%	0.947 (0.929−0.962)
DeepSeek-V3	NP	85.3%	86.5%	73.7%	93.0%	0.859 (0.834−0.884)
DeepSeek-V3	CGP	89.5%	96.2%	91.2%	95.4%	0.928 (0.907−0.947)
DeepSeek-R1	NP	91.0%	79.8%	66.7%	95.2%	0.854 (0.829−0.877)
DeepSeek-R1	CGP	95.1%	94.8%	89.0%	97.8%	0.950 (0.934−0.964)
Gemini-2.5Pro	NP	87.2%	76.4%	61.9%	93.1%	0.818 (0.790−0.844)
Gemini-2.5Pro	CGP	98.5%	91.2%	83.2%	99.3%	0.948 (0.935−0.961)

Notes:

- Best overall performance for each task highlighted in bold.
- PPV: Positive Predictive Value; NPV: Negative Predictive Value.
- 95% confidence intervals were calculated using bootstrap resampling (n=1000).

Table S6. Clinical Reasoning Fidelity Score (CRFS) Distribution (1–5)

A. CGP Condition

Model	N	1	2	3	4	5	≥4 (%)
DeepSeek-V3	296	16	32	8	23	217	81.1%
GLM-4.5	296	18	26	19	70	163	78.7%
DeepSeek-R1	296	11	16	36	124	109	78.7%
Qwen3-235B-Thinking	296	7	35	25	71	158	77.4%
Qwen3-235B-Instruct	296	11	42	16	61	166	76.7%
Gemini-2.5Pro	296	36	25	8	91	136	76.7%
Qwen-Next-80B	296	12	51	14	63	156	74.0%
Qwen3-8B	296	15	37	31	39	174	72.0%
GPT-OSS-120B	296	22	47	18	60	149	70.6%
MedGemma	296	17	34	62	92	91	61.8%

B. NP Condition

Model	N	1	2	3	4	5	≥4 (%)
DeepSeek-V3	296	24	33	52	97	90	63.2%
GLM-4.5	296	16	22	65	112	81	65.2%
DeepSeek-R1	296	14	45	58	82	97	60.5%
Qwen3-235B-Thinking	296	12	38	73	106	67	58.4%
Qwen3-235B-Instruct	296	17	42	56	91	90	61.1%
Gemini-2.5Pro	296	13	58	62	74	88	55.1%
Qwen-Next-80B	296	16	48	67	100	65	55.7%
Qwen3-8B	296	77	52	65	54	48	34.5%
GPT-OSS-120B	296	22	40	61	85	88	58.4%
MedGemma	296	98	72	51	48	27	25.3%

Notes:

- CRFS was scored on 150 patients (300 vessels).

Table S7. Human-AI Study Performance per Physician and Center A-only Sensitivity Analysis

A. Human–AI Study Performance per Physician

Physician	Level	Condition	N	Six-class agreement (%)	Macro F1	Weighted κ
Physician A	Junior	No AI	400	60.75%	0.465	0.026
Physician A	Junior	Qwen3-8B	400	83.75%	0.767	0.658
Physician A	Junior	DeepSeek-R1	400	87.25%	0.801	0.715
Physician B	Junior	No AI	400	62.75%	0.455	0.055
Physician B	Junior	Qwen3-8B	400	82.50%	0.747	0.549
Physician B	Junior	DeepSeek-R1	400	88.25%	0.827	0.713
Physician C	Senior	No AI	400	78.75%	0.772	0.614
Physician C	Senior	Qwen3-8B	400	86.75%	0.802	0.705
Physician C	Senior	DeepSeek-R1	400	90.75%	0.867	0.811
Physician D	Senior	No AI	400	75.25%	0.597	0.229
Physician D	Senior	Qwen3-8B	400	88.25%	0.867	0.740
Physician D	Senior	DeepSeek-R1	400	93.75%	0.923	0.861

Notes:

- Within-group variability was small across all conditions.
- For Junior physicians (A vs B), six-class agreement differences ranged from 1.0 to 2.0 percentage points.
- For Senior physicians (C vs D), differences ranged from 1.5 to 3.5 percentage points.
- These within-group variations were substantially smaller than between-group differences (Junior vs Senior: ~15 percentage points) and the AI assistance effect sizes (+21–26 percentage points for Junior; +10–15 percentage points for Senior), indicating consistent performance within experience levels.

B. Center A-only sensitivity analysis

Physician	Level	Condition	N vessels	Six-class agreement (%)
**Physician A**	**Junior**	**No AI**	**376**	**60.64%**
**Physician A**	**Junior**	**Qwen3-8B**	**376**	**84.04%**
**Physician A**	**Junior**	**DeepSeek-R1**	**376**	**86.97%**
**Physician B**	**Junior**	**No AI**	**376**	**64.10%**
**Physician B**	**Junior**	**Qwen3-8B**	**376**	**83.51%**
**Physician B**	**Junior**	**DeepSeek-R1**	**376**	**88.03%**
**Physician C**	**Senior**	**No AI**	**376**	**80.05%**
**Physician C**	**Senior**	**Qwen3-8B**	**376**	**86.97%**
**Physician C**	**Senior**	**DeepSeek-R1**	**376**	**90.96%**
**Physician D**	**Senior**	**No AI**	**376**	**75.00%**
**Physician D**	**Senior**	**Qwen3-8B**	**376**	**90.43%**
**Physician D**	**Senior**	**DeepSeek-R1**	**376**	**94.95%**
**Junior pooled**	**Junior**	**No AI**	**752**	**62.37%**
**Junior pooled**	**Junior**	**Qwen3-8B**	**752**	**83.78%**

Physician	Level	Condition	N vessels	Six-class agreement (%)
**Junior pooled**	**Junior**	**DeepSeek-R1**	**752**	**87.50%**
**Senior pooled**	**Senior**	**No AI**	**752**	**77.53%**
**Senior pooled**	**Senior**	**Qwen3-8B**	**752**	**88.70%**
**Senior pooled**	**Senior**	**DeepSeek-R1**	**752**	**92.95%**

Paired McNemar analyses in the Center A-only sensitivity analysis remained significant for all AI-assisted comparisons (all *p* < 0.001).

Table S8. Fixed-order reader Study Time and DAUS per Physician

A. Interpretation Time (s/case)

Physician	Level	Condition	N	Mean±SD (s)	Median (s)
Physician A	Junior	No AI	200	39.2±13.4	38.7
Physician A	Junior	Qwen3-8B	200	38.2±13.4	37.6
Physician A	Junior	DeepSeek-R1	200	57.2±17.0	54.3
Physician B	Junior	No AI	200	36.7±12.6	36.3
Physician B	Junior	Qwen3-8B	200	32.4±14.0	29.6
Physician B	Junior	DeepSeek-R1	200	60.2±16.2	60.5
Physician C	Senior	No AI	200	30.2±12.1	28.7
Physician C	Senior	Qwen3-8B	200	27.5±10.4	24.5
Physician C	Senior	DeepSeek-R1	200	58.2±17.3	55.3
Physician D	Senior	No AI	200	36.4±12.0	35.2
Physician D	Senior	Qwen3-8B	200	29.4±10.1	27.0
Physician D	Senior	DeepSeek-R1	200	64.2±27.5	57.1

B. Diagnostic Assistance Utility Score (DAUS, 1–5)

Physician	Level	AI	N	Median (IQR)	Mean±SD
Physician A	Junior	Qwen3-8B	400	4.0 (3.0-5.0)	3.88±1.32
Physician A	Junior	DeepSeek-R1	400	5.0 (4.0-5.0)	4.18±1.05
Physician B	Junior	Qwen3-8B	400	4.0 (4.0-5.0)	4.03±1.20
Physician B	Junior	DeepSeek-R1	400	4.0 (4.0-5.0)	4.14±1.09
Physician C	Senior	Qwen3-8B	400	4.0 (3.0-5.0)	3.81±1.36
Physician C	Senior	DeepSeek-R1	400	5.0 (3.0-5.0)	4.01±1.23
Physician D	Senior	Qwen3-8B	400	5.0 (4.0-5.0)	4.26±1.09
Physician D	Senior	DeepSeek-R1	400	5.0 (4.0-5.0)	4.33±0.99

Table S9. McNemar Test: LLM Models (CGP) vs Rule-based Baseline

Model	Model Agreement	Rule Agreement	b	c	χ^2	p-value
DeepSeek-R1	87.41%	50.92%	327	11	293.57	<0.0001
Qwen3-235B-Instruct	85.57%	50.92%	316	16	269.28	<0.0001
GLM-4.5	85.45%	50.92%	310	11	276.65	<0.0001
Gemini-2.5Pro	84.30%	50.92%	313	24	246.12	<0.0001
DeepSeek-V3	84.06%	50.92%	310	23	245.63	<0.0001
Qwen3-235B-Thinking	82.22%	50.92%	311	40	207.69	<0.0001
Qwen3-8B	81.52%	50.92%	293	28	217.12	<0.0001
Qwen-Next-80B	77.60%	50.92%	275	44	165.83	<0.0001
GPT-OSS-120B	73.44%	50.92%	259	64	116.52	<0.0001
MedGemma	72.29%	50.92%	239	54	115.55	<0.0001

Notes:

- McNemar test with continuity correction.
- b = cases where LLM correct but rule-based incorrect; c = cases where rule-based correct but LLM incorrect.
- **Confirmatory analysis** with Bonferroni correction for 10 comparisons (corrected $\alpha=0.005$); all comparisons remained significant after correction (all $p<0.0001$).
- Models are sorted by six-class agreement.

Table S10. McNemar Test: CGP vs NP Conditions

Model	CGP Agreement	NP Agreement	Δ	b	c	χ^2	p-value
Qwen3-8B	81.52%	38.91%	+42.6pp	396	27	320.15	<0.0001
MedGemma	72.29%	35.91%	+36.4pp	338	23	273.12	<0.0001
DeepSeek-R1	87.41%	71.25%	+16.2pp	185	45	84.00	<0.0001
Qwen-Next-80B	77.60%	64.09%	+13.5pp	205	88	45.92	<0.0001
Gemini-2.5Pro	84.30%	71.02%	+13.3pp	152	37	68.76	<0.0001
Qwen3-235B-Instruct	85.57%	72.40%	+13.2pp	175	61	54.11	<0.0001
DeepSeek-V3	84.06%	71.59%	+12.5pp	186	78	43.37	<0.0001
GLM-4.5	85.45%	73.90%	+11.5pp	156	56	46.23	<0.0001
GPT-OSS-120B	73.44%	63.16%	+10.3pp	180	91	28.58	<0.0001
Qwen3-235B-Thinking	82.22%	73.09%	+9.1pp	165	86	24.24	<0.0001

Notes:

- McNemar test with continuity correction.
- b = cases where CGP correct but NP incorrect; c = cases where NP correct but CGP incorrect.
- Δ = improvement in six-class agreement from NP to CGP.
- **Confirmatory analysis** with Bonferroni correction for 10 comparisons (corrected $\alpha=0.005$); all comparisons remained significant after correction (all $p<0.0001$).
- Models are sorted by improvement magnitude.

Table S11. McNemar Test: Pairwise Model Comparisons Matrix (CGP Condition)

Models are sorted by six-class agreement (descending). Each cell shows p-value with significance markers. Upper triangle: row model vs column model.

Model	DeepSeek-R1	Qwen3-235B-Instruct	GLM-4.5	Gemini-2.5 Pro	DeepSeek-V3	Qwen3-235B-Thinking	Qwen3-8B	Qwen-Next-80B	GPT-OSS-120B	MedGemma
DeepSeek-R1 (87.4%)	–	0.089	0.057	0.009	0.003	<.001	<.001	<.001	<.001	<.001
Qwen3-235B-Instruct (85.6%)		–	1.00	0.300	0.187	0.007	0.011	<.001	<.001	<.001
GLM-4.5 (85.5%)			–	0.382	0.230	0.014	0.014	<.001	<.001	<.001
Gemini-2.5Pro (84.3%)				–	0.925	0.114	0.109	<.001	<.001	<.001
DeepSeek-V3 (84.1%)					–	0.198	0.113	<.001	<.001	<.001
Qwen3-235B-Thinking (82.2%)						–	0.737	0.003	<.001	<.001

Qwen3-8B (81.5%)	–	0.021	<.001	<.001
Qwen-Next-80B (77.6%)		–	0.007	0.004
GPT-OSS-120B (73.4%)			–	0.588
MedGemma (72.3%)				–

Notes:

- **Exploratory analysis**; p-values are nominal (uncorrected).
- McNemar test with continuity correction (df=1).
- Models are sorted by six-class agreement.
- These pairwise comparisons are provided for descriptive purposes; readers should focus on effect sizes (six-class agreement differences) rather than statistical significance.

Table S12. Reader Study Paired Agreement Analyses

A. Group-Level Vessel-Level Planned Contrasts

Comparison	Agreement	OR (95% CI)	Nominal p	Holm p	Inference
Junior: No AI vs Qwen3-8B	61.8% vs 83.1%	3.06 (2.50-3.75)	<0.001	<0.001	Significant
Junior: Qwen3-8B vs DeepSeek-R1	83.1% vs 87.8%	1.45 (1.24-1.71)	<0.001	<0.001	Significant
Senior: No AI vs Qwen3-8B	77.0% vs 87.5%	2.09 (1.58-2.77)	<0.001	<0.001	Significant
Senior: Qwen3-8B vs DeepSeek-R1	87.5% vs 92.2%	1.70 (1.41-2.06)	<0.001	<0.001	Significant
Baseline No AI: Junior vs Senior	61.8% vs 77.0%	2.08 (1.68-2.57)	<0.001	<0.001	Significant

B. Reader-Specific Vessel-Level Secondary Analyses

Physician	Level	Comparison	Agreement	Improved	Worsened	Nominal p	Holm p	Inference
Physician A	Junior	No AI vs Qwen3-8B	60.8% vs 83.8%	113	21	<0.001	<0.001	Significant
Physician B	Junior	No AI vs Qwen3-8B	62.7% vs 82.5%	108	29	<0.001	<0.001	Significant
Physician C	Senior	No AI vs Qwen3-8B	78.8% vs 86.8%	61	29	<0.001	<0.001	Significant
Physician D	Senior	No AI vs Qwen3-8B	75.2% vs 88.2%	86	34	<0.001	<0.001	Significant
Physician A	Junior	Qwen3-8B vs DeepSeek-R1	83.8% vs 87.2%	16	2	0.001	0.002	Significant
Physician B	Junior	Qwen3-8B vs DeepSeek-R1	82.5% vs 88.2%	34	11	<0.001	0.002	Significant
Physician C	Senior	Qwen3-8B vs	86.8% vs	16	0	<0.001	<0.001	Significant

		DeepSeek-R1	90.8%					
Physician D	Senior	Qwen3-8B vs DeepSeek-R1	88.2% vs 93.8%	25	3	<0.001	<0.001	Significant

C. Group-Level Patient-Level Planned Contrasts

**Comparison**	**Agreement**	**OR (95% CI)**	**Nominal p**	**Holm p**	**Inference**
Junior: No AI vs Qwen3-8B	41.0% vs 71.0%	3.52 (2.68-4.63)	<0.001	<0.001	Significant
Junior: Qwen3-8B vs DeepSeek-R1	71.0% vs 78.8%	1.51 (1.27-1.80)	<0.001	<0.001	Significant
Senior: No AI vs Qwen3-8B	59.8% vs 80.2%	2.74 (1.98-3.78)	<0.001	<0.001	Significant
Senior: Qwen3-8B vs DeepSeek-R1	80.2% vs 87.8%	1.76 (1.43-2.18)	<0.001	<0.001	Significant
Baseline No AI: Junior vs Senior	41.0% vs 59.8%	2.14 (1.63-2.81)	<0.001	<0.001	Significant

D. Reader-Specific Patient-Level Secondary Analyses

**Physician**	**Level**	**Comparison**	**Agreement**	**Improved**	**Worsened**	**Nominal p**	**Holm p**	**Inference**
Physician A	Junior	No AI vs Qwen3-8B	39.5% vs 73.0%	84	17	<0.001	<0.001	Significant
Physician B	Junior	No AI vs Qwen3-8B	42.5% vs 69.0%	76	23	<0.001	<0.001	Significant
Physician C	Senior	No AI vs Qwen3-8B	62.0% vs 79.0%	52	18	<0.001	<0.001	Significant
Physician D	Senior	No AI vs	57.5% vs	70	22	<0.001	<0.001	Significant

		Qwen3-8B	81.5%					
Physician A	Junior	Qwen3-8B vs DeepSeek-R1	73.0% vs 79.0%	14	2	0.004	0.004	Significant
Physician B	Junior	Qwen3-8B vs DeepSeek-R1	69.0% vs 78.5%	27	8	0.002	0.004	Significant
Physician C	Senior	Qwen3-8B vs DeepSeek-R1	79.0% vs 86.0%	14	0	<0.001	<0.001	Significant
Physician D	Senior	Qwen3-8B vs DeepSeek-R1	81.5% vs 89.5%	19	3	<0.001	0.003	Significant

Notes:

- Vessel-level analyses treated the left and right carotid arteries as separate observations. Group-level inference used logistic GEE with patient-level clustering; within-group method contrasts additionally adjusted for physician and vessel side.
- Patient-level agreement was defined as correct classification of both carotid arteries for a given patient. Patient-level group inference used logistic GEE with patient-level clustering; within-group method contrasts additionally adjusted for physician.
- Reader-specific paired comparisons used exact McNemar tests. Agreement values are reported in the same order as the comparison labels. Improved denotes cases classified correctly under the second-listed condition but incorrectly under the first-listed condition; Worsened denotes the reverse.
- Holm correction was applied within the five planned group-level contrasts separately for vessel-level and patient-level analyses. Reader-specific secondary analyses were Holm-adjusted within each endpoint/comparison family across the four physicians.

Table S13. Human-AI Study Binary Classification Performance

To validate clinical applicability, we regrouped six-class predictions into two binary tasks: (1) Advanced Lesion Detection (Normal/I-II vs III-VIII) for screening patients requiring monitoring; (2) Complex Plaque Detection (Type VI vs non-VI) for identifying high stroke risk.

A. Advanced Lesion Detection (Normal/I-II vs III-VIII)

Physician	Level	Condition	N	Agreement	Sensitivity	Specificity	F1	AUC	Kappa
Physician A	Junior	No AI	400	96.5%	96.5%	96.6%	0.975	0.965	0.917
Physician A	Junior	Qwen3-8B	400	99.2%	99.6%	98.3%	0.995	0.990	0.982
Physician A	Junior	DeepSeek-R1	400	99.2%	99.6%	98.3%	0.995	0.990	0.982
Physician B	Junior	No AI	400	96.5%	99.3%	89.8%	0.976	0.946	0.914
Physician B	Junior	Qwen3-8B	400	97.5%	99.3%	93.2%	0.982	0.963	0.939
Physician B	Junior	DeepSeek-R1	400	98.2%	99.6%	94.9%	0.988	0.973	0.957
Physician C	Senior	No AI	400	99.5%	100.0%	98.3%	0.996	0.992	0.988
Physician C	Senior	Qwen3-8B	400	98.0%	98.6%	96.6%	0.986	0.976	0.952
Physician C	Senior	DeepSeek-R1	400	98.5%	98.9%	97.5%	0.989	0.982	0.964
Physician D	Senior	No AI	400	98.8%	99.3%	97.5%	0.991	0.984	0.970
Physician D	Senior	Qwen3-8B	400	99.0%	99.6%	97.5%	0.993	0.986	0.976

Physician D	Senior	DeepSeek-R1	400	99.5%	100.0%	98.3%	0.996	0.992	0.988

Summary by Experience Level:

Group	Condition	Agreement	Sensitivity	Specificity	F1
Junior (n=2)	No AI	96.5±0.0%	97.9%	93.2%	0.975
Junior (n=2)	Qwen3-8B	98.4±0.9%	99.5%	95.8%	0.989
Junior (n=2)	DeepSeek-R1	98.8±0.5%	99.6%	96.6%	0.991
Senior (n=2)	No AI	99.1±0.4%	99.6%	97.9%	0.994
Senior (n=2)	Qwen3-8B	98.5±0.5%	99.1%	97.0%	0.989
Senior (n=2)	DeepSeek-R1	99.0±0.5%	99.5%	97.9%	0.993
Overall (n=4)	No AI	97.8±1.3%	98.8%	95.6%	0.985
Overall (n=4)	Qwen3-8B	98.4±0.7%	99.3%	96.4%	0.989
Overall (n=4)	DeepSeek-R1	98.9±0.5%	99.6%	97.2%	0.992

B. Complex Plaque Detection (Type VI vs Non-VI)

Physician	Level	Condition	N	Agreement	Sensitivity	Specificity	F1	AUC	Kappa
Physician A	Junior	No AI	400	76.2%	53.4%	87.6%	0.599	0.705	0.434
Physician A	Junior	Qwen3-8B	400	91.2%	77.4%	98.1%	0.855	0.878	0.793
Physician A	Junior	DeepSeek-R1	400	93.8%	85.0%	98.1%	0.900	0.915	0.855
Physician B	Junior	No AI	400	86.8%	68.4%	95.9%	0.774	0.822	0.683

Physician B	Junior	Qwen3-8B	400	93.2%	90.2%	94.8%	0.899	0.925	0.848
Physician B	Junior	DeepSeek-R1	400	95.2%	92.5%	96.6%	0.928	0.946	0.893
Physician C	Senior	No AI	400	86.0%	60.2%	98.9%	0.741	0.795	0.652
Physician C	Senior	Qwen3-8B	400	92.5%	81.2%	98.1%	0.878	0.897	0.824
Physician C	Senior	DeepSeek-R1	400	94.2%	85.0%	98.9%	0.908	0.919	0.866
Physician D	Senior	No AI	400	93.2%	81.2%	99.3%	0.889	0.902	0.841
Physician D	Senior	Qwen3-8B	400	92.5%	85.7%	95.9%	0.884	0.908	0.828
Physician D	Senior	DeepSeek-R1	400	96.5%	92.5%	98.5%	0.946	0.955	0.920

Summary by Experience Level:

Group	Condition	Agreement	Sensitivity	Specificity	F1
Junior (n=2)	No AI	81.5±5.3%	60.9%	91.8%	0.687
Junior (n=2)	Qwen3-8B	92.2±1.0%	83.8%	96.4%	0.877
Junior (n=2)	DeepSeek-R1	94.5±0.8%	88.7%	97.4%	0.914
Senior (n=2)	No AI	89.6±3.6%	70.7%	99.1%	0.815
Senior (n=2)	Qwen3-8B	92.5±0.0%	83.5%	97.0%	0.881
Senior (n=2)	DeepSeek-R1	95.4±1.1%	88.7%	98.7%	0.927
Overall (n=4)	No AI	85.6±6.1%	65.8%	95.4%	0.751

Overall (n=4)	Qwen3-8B	92.4±0.7%	83.6%	96.7%	0.879
Overall (n=4)	DeepSeek-R1	94.9±1.1%	88.7%	98.0%	0.921

C. McNemar Test for Binary Classification Tasks

Advanced Lesion Detection:

Physician	Comparison	b	c	χ^2	p-value
Physician A	No AI vs DeepSeek-R1	1	12	7.69	0.0055
Physician B	No AI vs Qwen3-8B	8	12	0.45	0.5023
Physician B	No AI vs DeepSeek-R1	6	13	1.89	0.1687
Physician C	No AI vs Qwen3-8B	8	2	2.50	0.1138
Physician C	No AI vs DeepSeek-R1	6	2	1.12	0.2888
Physician D	No AI vs Qwen3-8B	4	5	0.00	1.0000
Physician D	No AI vs DeepSeek-R1	2	5	0.57	0.4497

Complex Plaque Detection:

Physician	Comparison	b	c	χ^2	p-value
Physician A	No AI vs Qwen3-8B	13	73	40.48	<0.0001
Physician A	No AI vs DeepSeek-R1	9	79	54.10	<0.0001
Physician A	Qwen3-8B vs DeepSeek-R1	0	10	8.10	0.0044
Physician B	No AI vs Qwen3-8B	22	48	8.93	0.0028
Physician B	No AI vs DeepSeek-R1	14	48	17.56	<0.0001

Physician C	No AI vs Qwen3-8B	9	35	14.20	0.0002
Physician C	No AI vs DeepSeek-R1	7	40	21.79	<0.0001
Physician C	Qwen3-8B vs DeepSeek-R1	0	7	5.14	0.0233
Physician D	No AI vs Qwen3-8B	26	23	0.08	0.7751
Physician D	No AI vs DeepSeek-R1	11	24	4.11	0.0425
Physician D	Qwen3-8B vs DeepSeek-R1	1	17	12.50	0.0004

Notes:

- Binary classification tasks were derived from six-class predictions.
- Advanced Lesion Detection: Normal/I-II (negative) vs III-VIII (positive).
- Complex Plaque Detection: Non-VI (negative) vs VI (positive).
- For Advanced Lesion Detection, baseline performance was already high (>96%), limiting room for AI-assisted improvement.
- For Complex Plaque Detection, AI assistance showed substantial and statistically significant improvements, particularly for junior physicians (sensitivity improved from 60.9% to 88.7%).
- McNemar test with continuity correction; b = cases where first condition correct but second incorrect; c = cases where second condition correct but first incorrect.

Table S14. Macro F1 vs Weighted F1 Comparison (CGP Condition)

Model	Agreement	Macro F1	Weighted F1	Δ (Weighted − Macro)
DeepSeek-R1	87.41%	0.841	0.868	+0.026
Qwen3-235B-Instruct	85.57%	0.774	0.852	+0.078
GLM-4.5	85.45%	0.767	0.849	+0.082
Gemini-2.5Pro	84.30%	0.770	0.836	+0.066
DeepSeek-V3	84.06%	0.765	0.839	+0.074
Qwen3-235B-Thinking	82.22%	0.736	0.821	+0.085
Qwen3-8B	81.52%	0.717	0.824	+0.107
Qwen-Next-80B	77.60%	0.671	0.771	+0.100
GPT-OSS-120B	73.44%	0.671	0.743	+0.072
MedGemma	72.29%	0.560	0.711	+0.151
Rule-based	50.92%	0.286	0.432	+0.146

Notes:

- Weighted F1 assigns class weights proportional to sample frequency, while Macro F1 treats all classes equally.
- The positive Δ indicates that all models perform relatively better on high-frequency classes (Normal, VI) than on low-frequency classes (III, VII).
- DeepSeek-R1 shows the smallest gap (+0.026), suggesting the most balanced performance across all phenotype categories.
- Models with larger gaps (e.g., MedGemma +0.151, Qwen3-8B +0.107) show relatively weaker performance on rare phenotypes.

Table S15. Qwen3-8B Output Stability and Chinese-English Agreement

Analysis	Prompting condition	Statistic	Value	Interpretation
Repeated-run stability	CGP	Fleiss' kappa	0.9050	High agreement across three repeated CGP runs
Repeated-run stability	NP	Fleiss' kappa	0.8477	High agreement across three repeated NP runs
Chinese-English agreement	CGP	Cohen's kappa	0.8579	Higher Chinese-English agreement under CGP
Chinese-English agreement	NP	Cohen's kappa	0.7318	Substantial Chinese-English agreement under NP

Notes:

- Repeated-run stability was assessed by running Qwen3-8B three times under the same prompting condition and quantifying inter-run agreement with Fleiss' kappa.
- Chinese-English agreement was assessed by comparing Qwen3-8B outputs from Chinese report inputs and English-translated report inputs using Cohen's kappa.
- These analyses evaluate output stability and language-transfer consistency in the report-based text-to-label mapping task; they do not replace external validation on independently authored non-Chinese reports.

Table S16. Post hoc sensitivity analysis

A. Minimum detectable differences in agreement at 80% power

Analysis level	No. of observational units	Proportion of discordant pairs	No. of discordant pairs	Minimum detectable p_{improved}	Minimum detectable Δ agreement
Vessel	400	20%	80	0.651	6.0 pp
Vessel	400	25%	100	0.635	6.8 pp
Vessel	400	30%	120	0.625	7.5 pp
Vessel	400	35%	140	0.617	8.2 pp
Patient	200	35%	70	0.655	10.8 pp
Patient	200	40%	80	0.651	12.1 pp
Patient	200	45%	90	0.637	12.3 pp
Patient	200	50%	100	0.635	13.5 pp

Notes:

- Vessel level: The observed discordance ranged from 22.5% to 34.2% (90–137 pairs), corresponding to a minimum detectable Δ of approximately 6–8 percentage points.
- Patient level: The observed discordance ranged from 35.0% to 50.5% (70–101 pairs), corresponding to a minimum detectable Δ of approximately 11–14 percentage points.

B.Observed versus minimum detectable differences in agreement

Reader	Experience	Analysis level	Baseline agreement	AI-assisted agreement	Observed Δ	Minimum detectable Δ	Exceeds MDE
Physician A	Junior	Vessel	60.8%	83.8%	23.0 pp	~7.6 pp	Yes
Physician B	Junior	Vessel	62.8%	82.5%	19.7 pp	~7.9 pp	Yes
Physician C	Senior	Vessel	78.8%	86.8%	8.0 pp	~6.2 pp	Yes

Reader	Experience	Analysis level	Baseline agreement	AI-assisted agreement	Observed Δ	Minimum detectable Δ	Exceeds MDE
Physician D	Senior	Vessel	75.3%	88.3%	13.0 pp	~7.5 pp	Yes
Physician A	Junior	Patient	39.5%	73.0%	33.5 pp	~13.1 pp	Yes
Physician B	Junior	Patient	42.5%	69.0%	26.5 pp	~13.0 pp	Yes
Physician C	Senior	Patient	62.0%	79.0%	17.0 pp	~10.8 pp	Yes
Physician D	Senior	Patient	57.5%	81.5%	24.0 pp	~12.4 pp	Yes

Notes:

- The observed effect sizes for all confirmatory comparisons exceeded the corresponding minimum detectable thresholds. At the vessel level, the minimum detectable Δ was 6–8 percentage points, whereas the observed effects were 8–23 percentage points. At the patient level, the minimum detectable Δ was 11–14 percentage points, whereas the observed effects were 17–34 percentage points.
- This study had limited power to detect smaller clinically meaningful effects, such as a 5-percentage-point improvement. Future studies aiming to detect more subtle improvements would require larger sample sizes.
- This sensitivity analysis was conducted post hoc and should not be considered a substitute for an a priori sample size calculation in a prospective study. Owing to the retrospective consecutive-cohort design, all eligible cases during the study period were included, and the sample size was determined by data availability rather than by prospective design.

Supplementary Figures

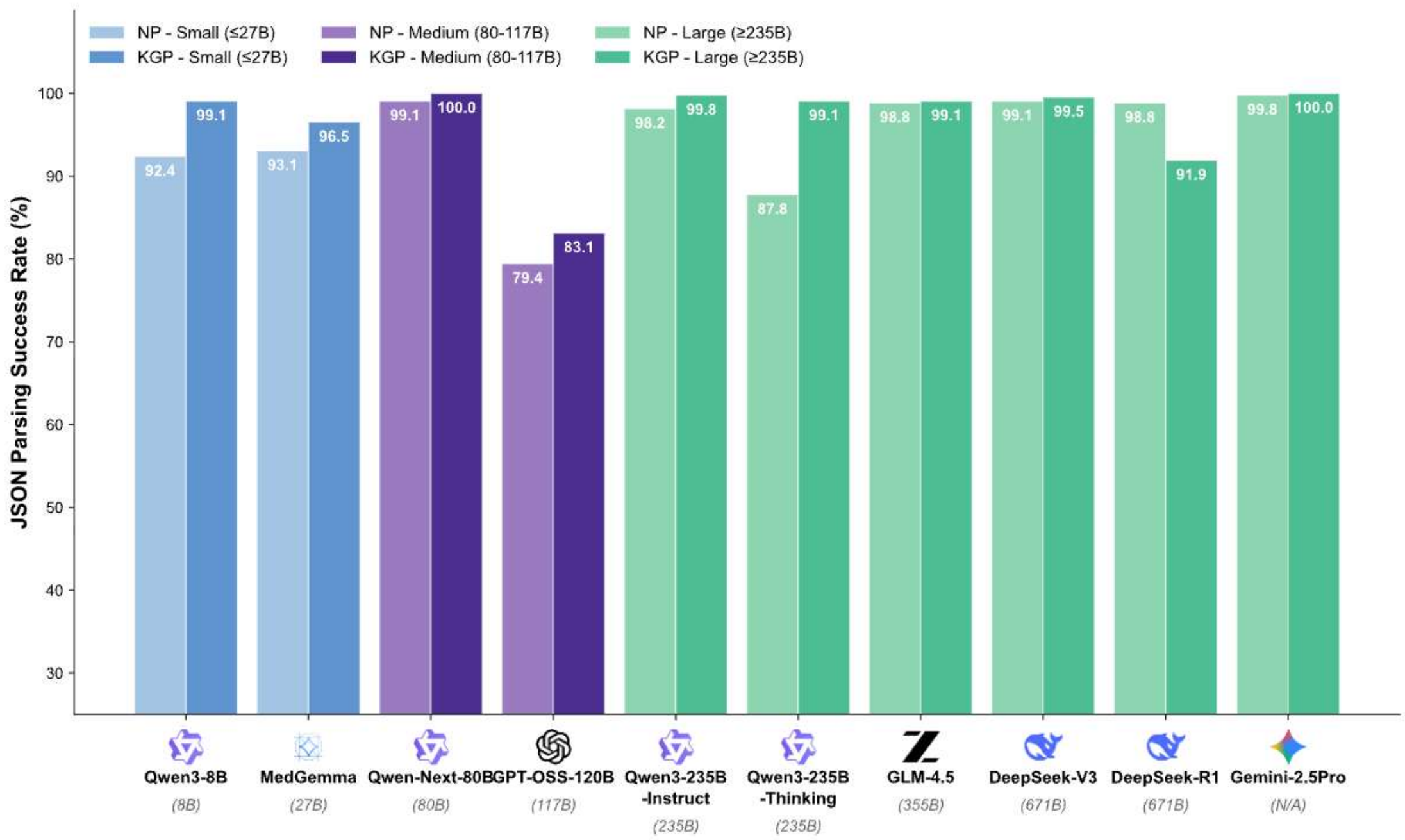

Figure S1. JSON Parsing Success Rate

This figure shows the JSON parsing success rate for each LLM model across both NP and CGP conditions. All models achieved >95% valid JSON output rate, indicating reliable structured output generation.

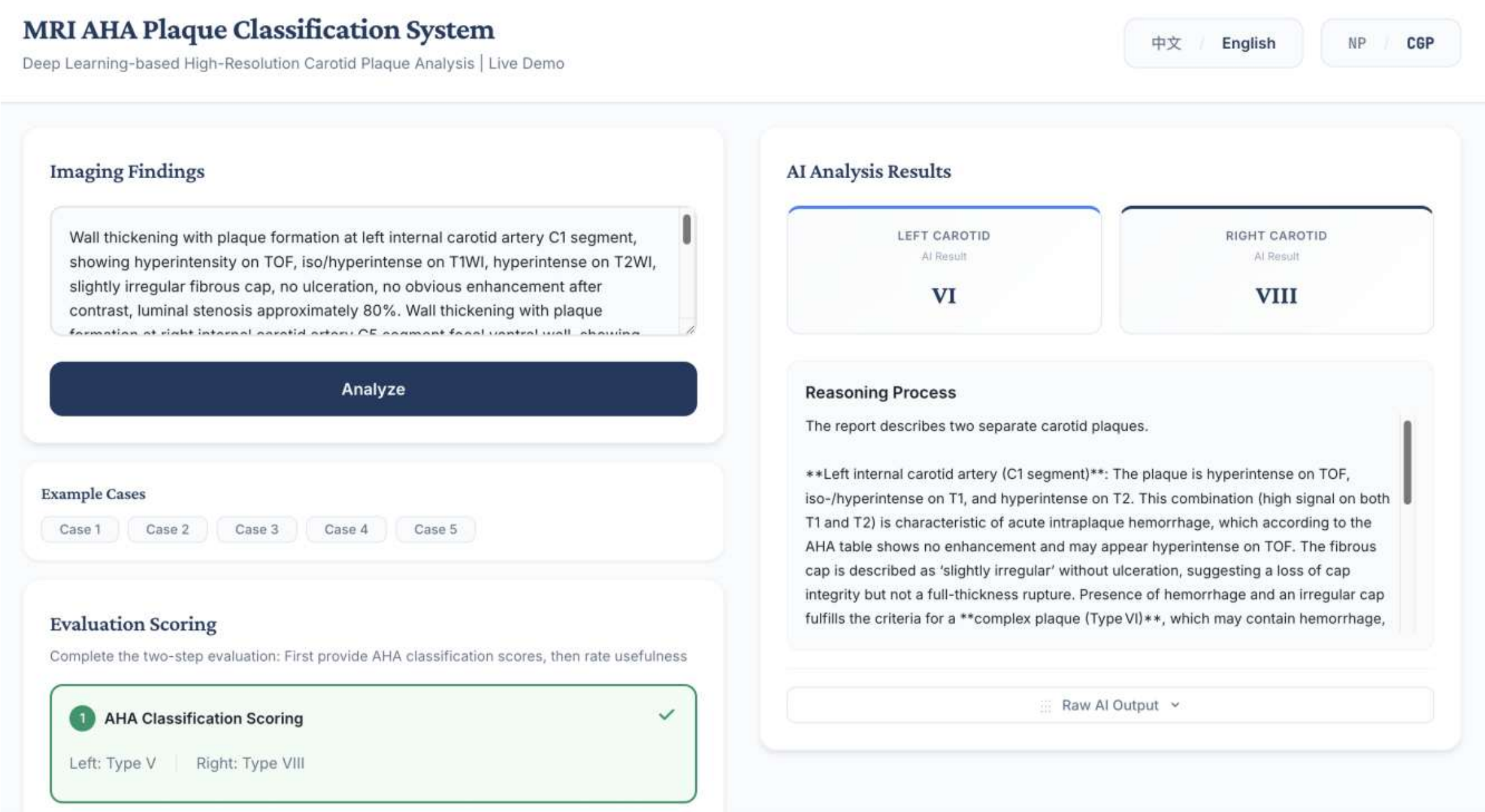

Figure S2. Web-Based Clinical Validation Platform

Web-based reader study interface for modified AHA classification of carotid plaques from free-text HRMRI reports. The system displays bilateral classification options and, when enabled, provides LLM-generated results with structured reasoning to support physician review. A representative demo is available at https://demo.scosine.org/. All reader study data remained on local servers without external API calls.

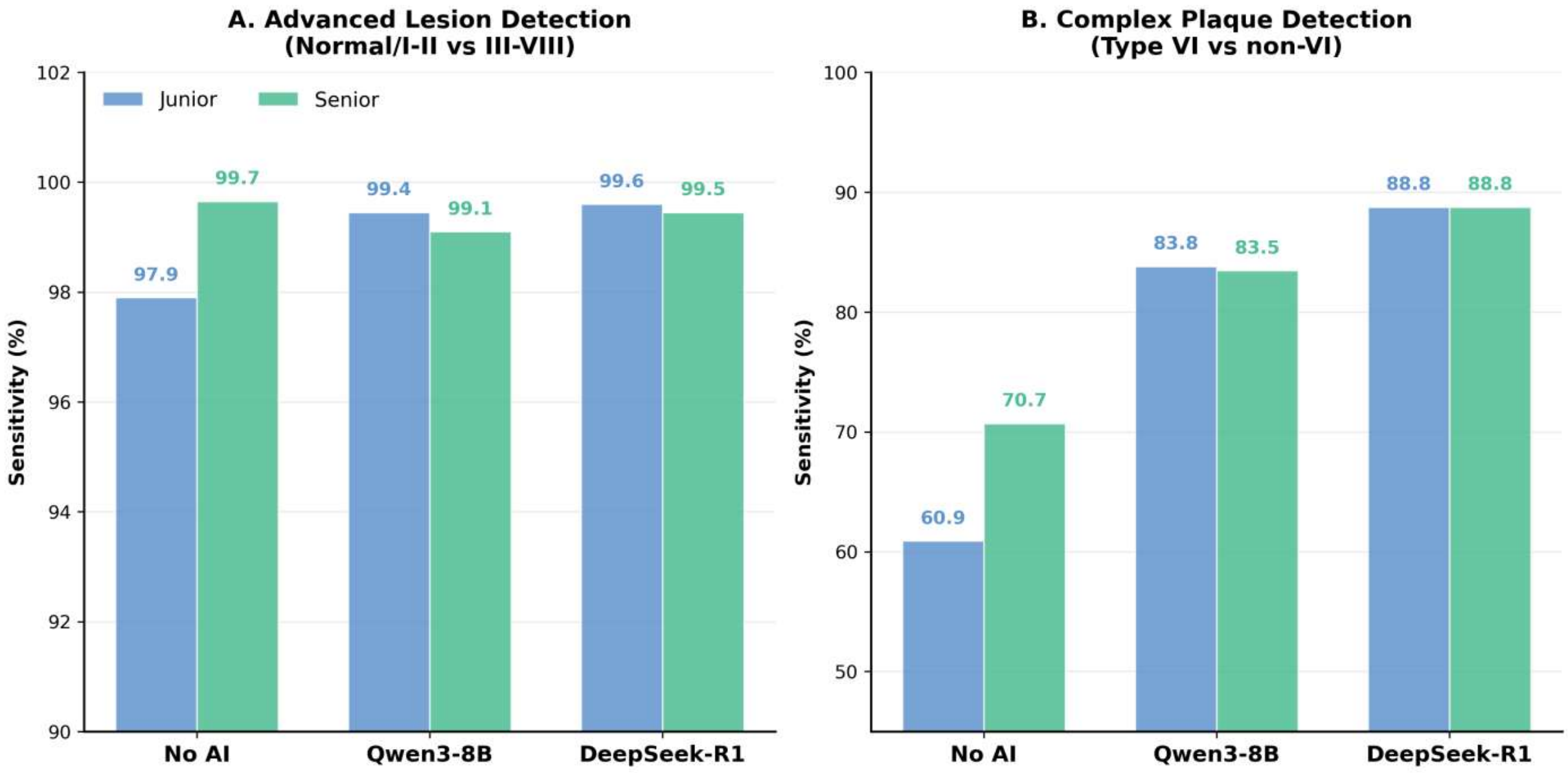

Figure S3. Binary Classification Performance in Human-AI Study

Binary classification performance in the MRMC human–AI study. Panel A shows advanced lesion detection (Normal/I-II vs III–VIII), with high baseline performance and limited room for further improvement. Panel B shows complex plaque detection (Type VI vs non-VI), where AI assistance substantially improved sensitivity, especially for junior physicians; with DeepSeek-R1, junior sensitivity increased from 60.9% to 88.7%, approaching senior-level performance. Error bars indicate 95% confidence intervals. Detailed results are provided in Table S13.

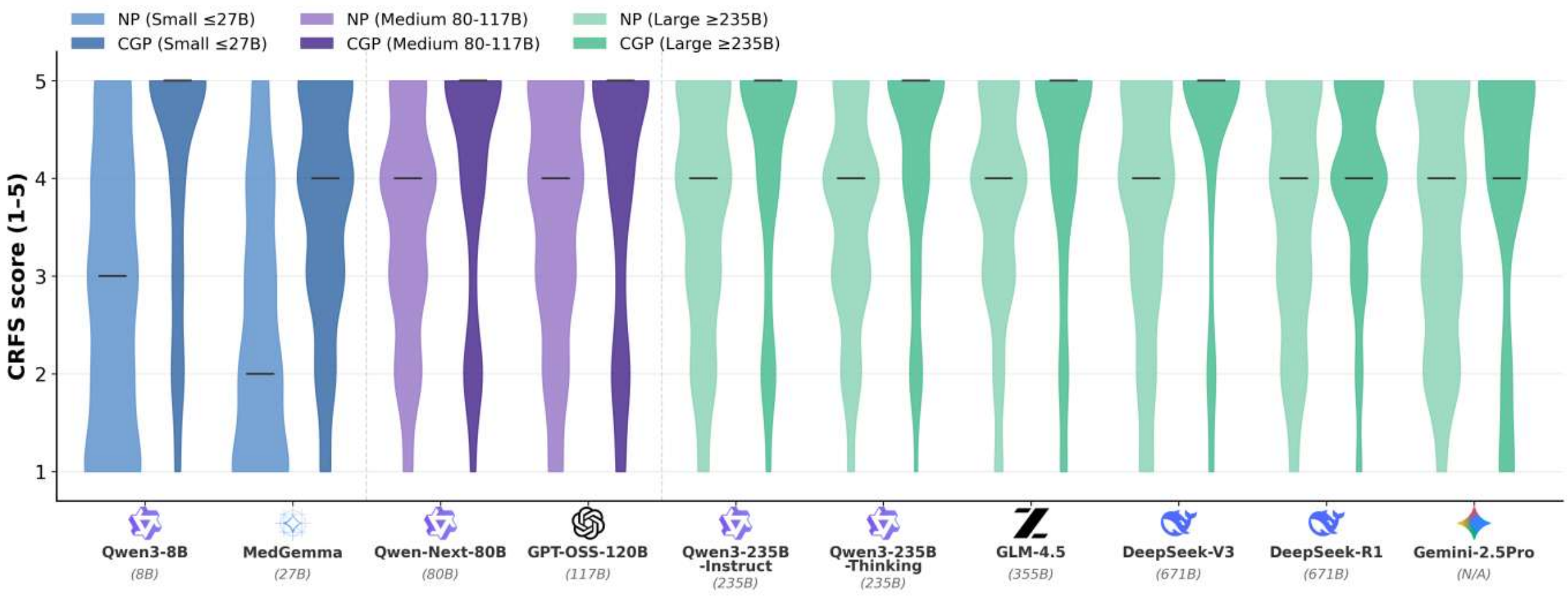

Figure S4. Clinical Reasoning Fidelity Score (CRFS) distributions

Violin plots displaying CRFS distributions (1–5 scale) for each model under NP and CGP conditions. Higher scores indicate better alignment with expert clinical logic.

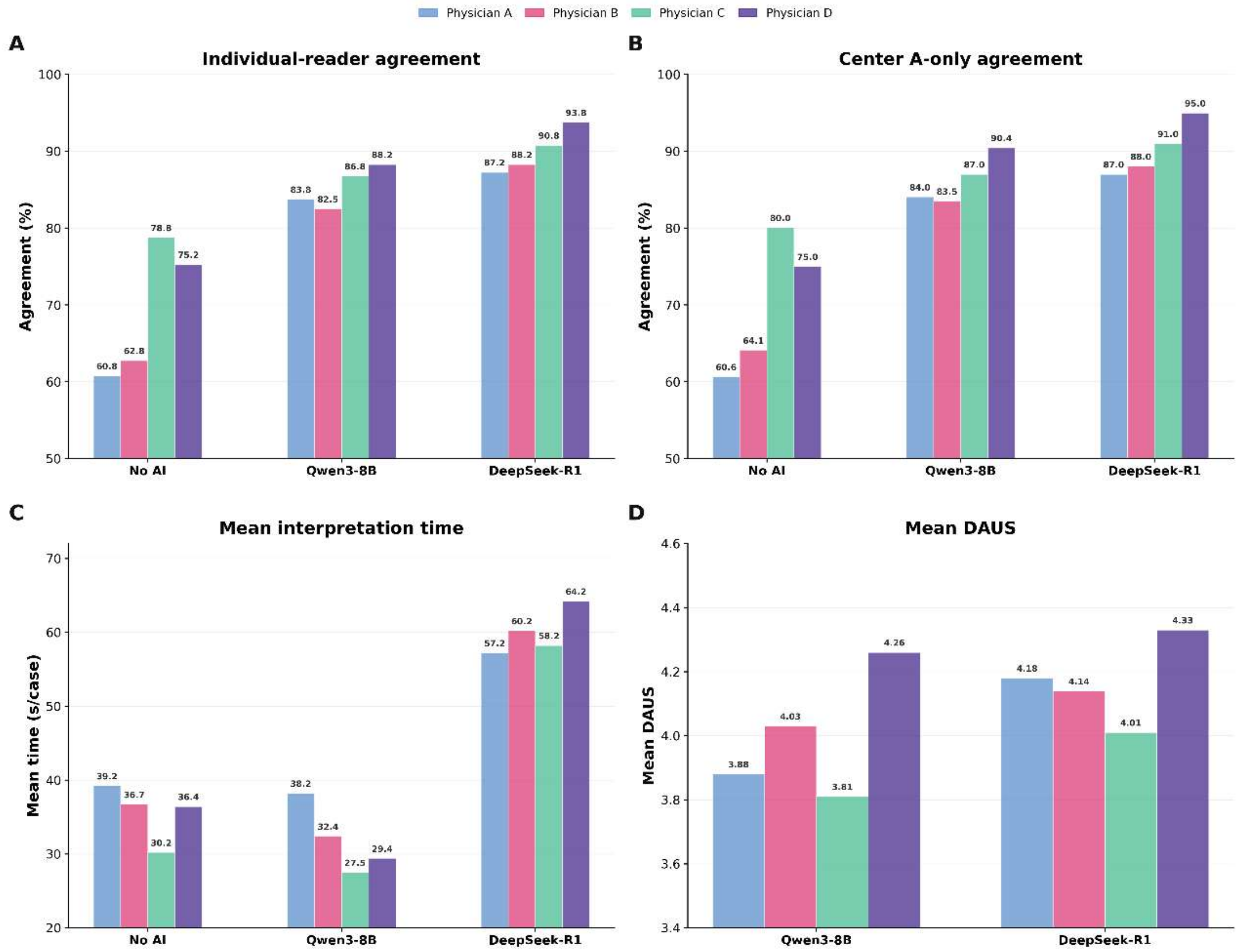

Figure S5. Reader Performance Summary

Individual-reader results are shown for six-class agreement in the full reader-study sample (A), Center A-only agreement sensitivity analysis (B), mean interpretation time per case (C), and mean Diagnostic Assistance Utility Score (DAUS) under AI-assisted conditions (D). Bars represent individual readers under each reading condition.
